# The round goby genome provides insights into mechanisms that may facilitate biological invasions

**DOI:** 10.1186/s12915-019-0731-8

**Published:** 2020-01-28

**Authors:** Irene Adrian-Kalchhauser, Anders Blomberg, Tomas Larsson, Zuzana Musilova, Claire R. Peart, Martin Pippel, Monica Hongroe Solbakken, Jaanus Suurväli, Jean-Claude Walser, Joanna Yvonne Wilson, Magnus Alm Rosenblad, Demian Burguera, Silvia Gutnik, Nico Michiels, Mats Töpel, Kirill Pankov, Siegfried Schloissnig, Sylke Winkler

**Affiliations:** 1 Program Man-Society-Environment, Department of Environmental Sciences, University of Basel, Vesalgasse 1, 4051 Basel, Switzerland; 2 University of Bern, Institute for Fish and Wildlife Health, Länggassstrasse 122, 3012 Bern, Austria; 3 Department of Chemistry and Molecular Biology, University of Gothenburg, Medicinaregatan 9C, 41390 Gothenburg, Sweden; 4 Department of Marine Sciences, University of Gothenburg, Medicinaregatan 9C, 41390 Gothenburg, Sweden; 5 Department of Zoology, Charles University, Vinicna 7, 12844 Prague, Czech Republic; 6 Division of Evolutionary Biology, Faculty of Biology, Ludwig-Maximilians-Universität München, Grosshaderner Strasse 2, 82152 Planegg-, Martinsried, Germany; 7 Max Planck Institute of Molecular Cell Biology and Genetics, Pfotenhauerstrasse 108, 01307 Dresden, Germany; 8 Centre for Ecological and Evolutionary Synthesis, University of Oslo, Blindernveien 31, 0371 Oslo, Norway; 9 Institute for Genetics, University of Cologne, Zülpicher Strasse 47a, 50674 Köln, Germany; 10 Genetic Diversity Centre, ETH, Universitätsstrasse 16, 8092 Zurich, Switzerland; 11 Department of Biology, McMaster University, 1280 Main Street West, Hamilton, ON Canada; 12 NBIS Bioinformatics Infrastructure for Life Sciences, University of Gothenburg, Medicinaregatan 9C, 41390 Gothenburg, Sweden; 13 Biocenter, University of Basel, Klingelbergstrasse 50/70, 4056 Basel, Switzerland; 14 Institute of Evolution and Ecology, University of Tuebingen, Auf der Morgenstelle 28, 72076 Tübingen, Germany; 15 Research Institute of Molecular Pathology (IMP), Vienna BioCenter (VBC), 1030 Vienna, Austria

**Keywords:** PacBio, *Neogobius melanostomus*, Invasive species, Fish, Genomics, Evolution, Adaptation, Gene duplication, Vision, Olfaction, Innate immunity, Detoxification, Osmoregulation, Epigenetics

## Abstract

**Background:**

The invasive benthic round goby (*Neogobius melanostomus)* is the most successful temperate invasive fish and has spread in aquatic ecosystems on both sides of the Atlantic. Invasive species constitute powerful in situ experimental systems to study fast adaptation and directional selection on short ecological timescales and present promising case studies to understand factors involved the impressive ability of some species to colonize novel environments. We seize the unique opportunity presented by the round goby invasion to study genomic substrates potentially involved in colonization success.

**Results:**

We report a highly contiguous long-read-based genome and analyze gene families that we hypothesize to relate to the ability of these fish to deal with novel environments. The analyses provide novel insights from the large evolutionary scale to the small species-specific scale. We describe expansions in specific cytochrome P450 enzymes, a remarkably diverse innate immune system, an ancient duplication in red light vision accompanied by red skin fluorescence, evolutionary patterns of epigenetic regulators, and the presence of osmoregulatory genes that may have contributed to the round goby’s capacity to invade cold and salty waters. A recurring theme across all analyzed gene families is gene expansions.

**Conclusions:**

The expanded innate immune system of round goby may potentially contribute to its ability to colonize novel areas. Since other gene families also feature copy number expansions in the round goby, and since other Gobiidae also feature fascinating environmental adaptations and are excellent colonizers, further long-read genome approaches across the goby family may reveal whether gene copy number expansions are more generally related to the ability to conquer new habitats in Gobiidae or in fish.

**Electronic supplementary material:**

The online version of this article (10.1186/s12915-019-0731-8) contains supplementary material, which is available to authorized users.

## Introduction

Since the beginning of global trade and the colonial period, hundreds of species have colonized territories outside their native range. A fraction of those species proliferates at the expense of native species and ecosystems, i.e., they are invasive. While invasive species present challenges for biodiversity and ecosystem conservation, they also constitute exciting eco-evolutionary models for survival in and adaptation to novel or changing environments [1–4].

**Fig. 1.**
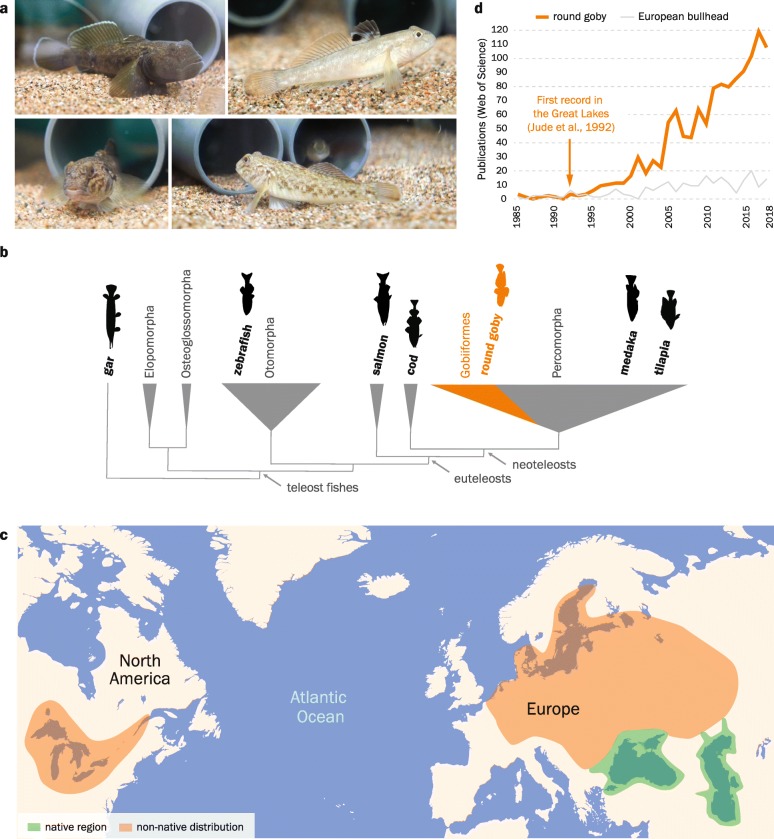
The round goby, an invasive benthic fish. **a** Wild-caught round goby in aquaria. Individuals usually feature a light gray or gray-brown mottled coloration, with a characteristic black dot on the first dorsal fin. Adults measure between 8 and 20 cm. During the reproductive season, males may become territorial and develop a black body color (first panel). **b** Phylogenetic position of the round goby among fishes. The round goby is a neoteleost and member of the Percomorpha. **c** Current geographical distribution of round goby. The round goby has spread from its native region (green) in the Ponto-Caspian area in Eurasia to many European rivers and lakes, the Baltic Sea, the Great Lakes and their tributaries (orange). **d** The growing relevance of the species as research model is reflected by increasing publication numbers. Web of Science records on round goby (orange) have risen since its first detection in the Great Lakes in contrast to records on a non-invasive fish with similar ecology (European bullhead, *Cottus gobio*; gray)

The benthic round goby *Neogobius melanostomus* (Fig. 1a) is a member of Percomorpha/Gobiiformes (Fig. 1b) and one of the most widespread invasive fish species. Since 1990, round gobies have been detected in over 20 countries outside their native Ponto-Caspian range. In some invaded regions of Europe and North America, they have become the most common fish species [5–7] (Fig. 1c). Lasting impacts on biodiversity and on ecosystems have been observed (see [8] for a summary of the impacts). In recent years, the round goby has therefore become a novel model for ecology, behavior, and evolution, which is reflected by rising publication numbers (Fig. 1d).

Genome analyses have previously provided significant insights into fish ecology and evolution. Examples are genome compaction [9], the transition from fin to limb [10], loss of major parts of adaptive immunity [11], or effects of genome duplication [12]. Since the round goby outcompetes and outperforms native and invasive benthic fish species with similar ecology [7, 13–15], the species is a promising candidate to study fundamental questions on the processes underlying exceptional performance of species in novel environments. Since round goby sequence data is presently quite restricted [16–22], relevant insights into round goby biology as well as starting points to study its impressive colonization ability are expected from the round goby genome sequence.

The survival of an individual in a novel environment may be influenced by its ability to perceive, react to, and accommodate to its new surroundings. In this study, we therefore explore the high-quality and contiguous genome assembly of the round goby for genes related to three categories: environmental perception, reaction to environmental conditions, and long-term accommodation to novel environments. We focus on gene families that have been hypothesized to play a role in the colonization of novel environments and on gene families relating to specific aspects of round goby invasion ecology.

For environmental perception, we investigated genes responsible for sensory perception in fishes. We specifically focused on the opsin genes for visual perception, as well as on the olfactory receptors for odor perception. Vision in fishes is often specifically adapted to environmental conditions, such as darkness in deep water [23], modified color spectrum in turbid water [24, 25], habitat color [26], or specific light regimes or light compositions [27–29]. The overall spectral sensitivity range of teleost fishes exceeds the human visual range and, in many cases, includes the UV [26] and far-red [30] spectrum. Similarly, olfaction is an essential chemoreception sense for fish, allowing for fast responses to predators and alarm cues, as well as for intra-species communication. Pheromones play an important role in the round goby [31–33], and males attract females into their nests by releasing them [34]. A particularly specialized sense of smell therefore may provide an advantage during initial population establishment in novel environments, when population densities are still low.

We further investigated genes that may mediate responses to novel environments, namely genes involved in detoxification, ion transport, and the immune system. The round goby occurs in chemically contaminated harbors [35–37] and appears to tolerate xenobiotic compounds well. This suggests that the round goby may be particularly well equipped to degrade and eliminate chemical pollutants. We therefore analyze the cytochrome P450 gene superfamily, which is a particularly important and conserved part of the vertebrate xenobiotic response [38]. The round goby is a euryhaline fish that tolerates a wide range of salinities (0 to 25 PSU / ‰) and temperatures (0–30 °C) and occurs at latitudes ranging from < 40° N in the Ponto-Caspian region to > 60° N in the Baltic Sea [39, 40]. We therefore study the genetic basis of osmoregulation and osmolyte production in round goby to gain insights into the evolution of salinity and cold tolerance and to possibly predict future range expansions. Finally, invasive species encounter an array of previously unknown pathogens when they colonize a habitat, and invasion success may be related to a species’ ability to tackle novel immune challenges [41]. Intriguingly, the round goby displays a low parasite load at the invasion front [42]. We therefore characterize key factors of the innate and the adaptive immune system.

We also investigated conserved gene regulators which might be involved in long-term adaptation to a novel environment. Mechanisms such as DNA methylation and histone modifications promote long- and short-term gene expression regulation and therefore mediate adaptations to altered conditions at the cellular level [43], but also regulate genome-scale evolutionary processes such as the distribution of meiotic recombination events [44] or transposon activity [45] and provide stochastic variability as basis for selection [46]. Epigenetic variants have been proposed to cause fitness-relevant differences in gene expression and phenotype [47, 48]. The ecological flexibility of the round goby has been linked to enhanced gene expression plasticity in response to environmental stimuli [49] and to their ability to pass information on water temperature to their offspring through maternal RNA [50]. To understand the features of core epigenetic regulators in the round goby, we focused on two widely conserved and well-characterized parts of the epigenetic machinery: the histone-methylating PRC2 complex and the DNA methylases. Both mechanisms are implicated in the regulation of developmental plasticity and gene expression and have been linked to plastic responses, behavioral changes, and environmental memory [51–55].

Finally, we take advantage of the high genome contiguity to investigate sex determination using RAD sequencing data. Fish display a wide variety of sex determination mechanisms, ranging from sex chromosomes to multilocus genetic sex determination to environmental sex determination [56], and sex determination in the round goby has not previously been investigated.

## Results

### The round goby genome

The round goby genome assembly (deposited at GenBank under the accession VHKM00000000; 57) consists of 1364 contigs with a total length of 1.00 Gb (1,003,738,563 bp), which is within the expected size range [57–59]. It is assembled to high contiguity (NG50 at 1,660,458 bp and N50 at 2,817,412 bp). GC content is 41.60%. An automated Maker gene annotation predicts a total of 38,773 genes and 39,166 proteins, of which 30,698 are longer than 100 amino acids see Table 1 for genome statistics; the annotation track is (available from the Zenodo data repository; 61). BUSCO analysis recovered 86.1% (eukaryota) and 86.9% (actinopterygia) complete BUSCOs for the assembly (see Table 1 for complete BUSCO results). The genome does not appear to contain a sex chromosome or a large sex-determining region, since a RAD-tag dataset from 40 females and 40 males (deposited at NCBI SRA as BioProject PRJNA547536; 62) with an estimated resolution of 25,000–45,000 bp does not contain any sex-specific loci.

**Table 1:** Statistics and resources

Assembly	
Number of contigs	1364
Total genome length (bp)	1,003,738,563
Longest contig (bp)	19,396,355
Smallest contig (bp)	21,178
N50 contig length (bp)	2,817,412
Annotation	
Number of genes	38,773
Genomic repeat content (%)	47
G + C (%)	41.60
LTR retrotransposons (%)	4.9–11.2
Eukaryote BUSCOs (*n* = 303)	Complete 86.1%, single-copy 82.8%, duplicated 3.3%, fragmented 4.6%, missing 9.3%
Actinopterygian BUSCOs (*n* = 4584)	Complete 85.4%, single-copy 82.0%, duplicated 3.4%, fragmented 8.2%, missing 6.4%
Accession	NCBI BioProject PRJNA549924 Accession VHKM00000000 [60]
Sequence data available in SRA	
RNA (Wellband & Heath 2017)	Liver transcriptome NCBI BioProject PRJNA321539 [61]
RNA (Adrian-Kalchhauser 2018)	Embryonic transcriptome (1–32 cell stages) from 16 clutches NCBI BioProject PRJNA547711 [62]
DNAme (Somerville 2019)	Brain DNA methylation data from 15 males NCBI BioProject PRJNA515617 [63]
RADseq (this study)	RAD Seq data from 120 individuals NCBI BioProject PRJNA547536 [63]
ATACseq (ongoing unpublished study headed by IAK)	ATAC Seq data of liver and brain from 50 individuals NCBI BioProject PRJNA551348 [64]

Key figures of the round goby genome assembly including annotation statistics and available short-read resources

Approximately 47% of the genome assembly is masked as various types of repetitive sequences by RepeatMasker in the Maker annotation pipeline. Upon closer analysis, the genome consists of approximately 9% predicted interspersed repeats, which is much lower than for zebrafish (*Danio rerio*, total genome size 1427.3 Mb, 46% predicted as interspersed repeats) but higher than for the more closely related threespine stickleback (*Gasterosteus aculeatus*, total genome size 446.6 Mb, 3.2% predicted as interspersed repeats). Among interspersed repeats, the long terminal repeat (LTR) retrotransposon family is the most common in many species including fish (Repbase, https://www.girinst.org/repbase/). RepeatMasker identifies 0.9% LTR retrotransposons in the round goby genome, but separately run de novo predictions with LTRfinder and LTRharvest indicate an underestimation of LTR retrotransposons and interspersed repeats by this approach estimate that the proportion of LTR retrotransposons in the round goby genome is 11.2% (3.8% LTRs with target-site-repeats; LTRfinder) or 4.9% (LTRharvest), respectively. Detailed results of repeat analyses are described in Additional file 1: Table S1.

The round goby genome sequence is currently complemented by a number of published and unpublished raw read resources. These resources include RNA sequencing data from early cleavage embryos [50] and adult liver [49], DNA methylation capture data from adult male brains [51], as well as unpublished RAD sequence tags derived from two Swiss populations, and ATAC seq reads from an ongoing study on brain and liver and are listed in Table 1.

### Sensory perception genes: vision

Vertebrates perceive color with cone cells expressing one of four types of opsin proteins (usually sensitive to the red, green, blue, and ultraviolet part of the spectrum) and dim light with rod cells expressing the rod opsin. The UV and blue light is detected by the short-wavelength sensitive SWS1 and SWS2 opsins, the green part of the spectrum is perceived mostly by the rhodopsin-like RH2 opsins, and the red color by the long-wavelength sensitive (LWS) opsins. Rod cells are active in the dim-light conditions and contain the rod opsin RH1 [65]. Gene duplications and losses of the opsin genes during fish evolution correlate to certain extent with adaptations to specific environments [23, 66].

**Fig. 2.**
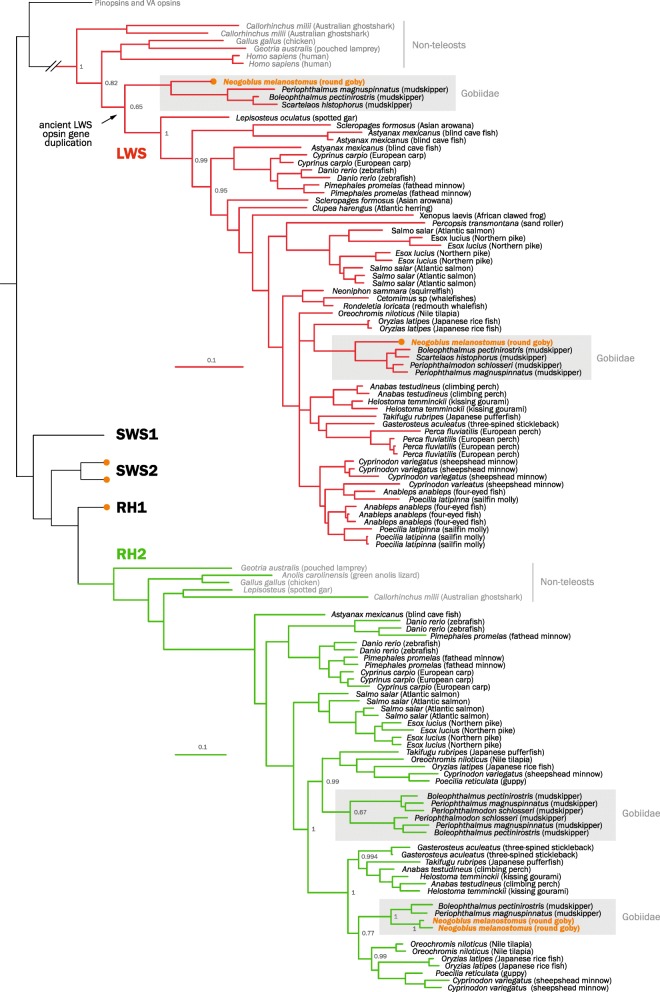
Phylogenetic tree of vertebrate opsin gene sequences. Maximum likelihood phylogenetic tree based on the cone and rod visual opsins and using VA opsins and pinopsins as outgroup. The round goby genome contains two LWS gene copies, which seem to be the results of an ancient gene duplication event, and two more recently duplicated RH2 gene copies. Round goby is indicated in orange. Red opsin branches are indicated in red; green opsin branches are indicated in green. Non-teleost species and the outgroup (VA opsins and pinopsins) are indicated in gray. Gray boxes highlight Gobiidae. See Additional file 2: Figure S1 for a tree including expanded SWS1, SWS2, and RH1 branches and Additional file 3: Figure S2 for an exon-based tree

We identified two cone opsin gene duplications in the round goby genome. Firstly, the genome features a recent duplication of the green-sensitive RH2 gene. RH2 duplications are a common phenomenon in fish (Fig. 2). Secondly, the genome features an ancient duplication of the long-wavelength red-sensitive LWS gene. The event can be traced most likely to the ancestor of all teleosts, or possibly even to the ancestor of Neopterygii (Fig. 2). As expected, the round goby genome further contains one dim-light rod opsin (RH1) gene and two blue-sensitive SWS2 genes [66] and, as previously reported for gobies, lacks the UV/violet-sensitive SWS1 gene [23, 28, 66] (see Additional file 2: Figure S1 for full tree including RH1, SWS1, and SWS2 branches).

The proposed ancestral position of the red opsin gene duplication is supported by three lines of evidence. First, the monophyly of all other teleost + gar LWS genes is strongly supported by the Bayesian analysis (Bayesian posterior probability value = 1). Second, the distant phylogenetic position is supported by trees based on individual exons, which indicate a low probability of a compromised phylogenetic signal, e.g., due to the partial gene conversion (see Additional file 3: Figure S2 for an exon-based tree). Three of four exons cluster at the same position as the whole gene, while the fourth exon (exon 4) cluster with the genes resulting from a more recent teleost-specific LWS duplication specific to *Astyanax* and *Scleropages* [67]. Third, the choice of outgroup (parietopsin or pinopsin) does not affect the position of the LWS2 gene. Together, these analyses suggest either (1) the presence of an ancient gene duplication event of the LWS gene in the ancestor of teleost and holostean fishes (i.e., *Neopterygii*) which was retained only in the goby family, or (2) a teleost-specific event, possibly identical to that reported for characins and bony tongues [67], with a subsequent concerted goby-specific sequence diversification in exons 2, 3, and 5.

The spectral sensitivity of photopigments, i.e., their excitation wavelength can be modified by substitutions in certain key amino acids [68]. We find that round goby LWS1 and LWS2 differ in the key spectral tuning site at amino acid 277 (position 261 of bovine rhodopsin, Table 2) suggesting a predicted but unverified sensitivity shift between the two genes of 10 nm.

**Table 2:** Opsin key residues

Species	Ecology	Gene	Key tuning amino acid site in round goby					Max. spectral sensitivity (wavelength)	Reference
			180	197	277	285	308		
		*Bovine rhodopsin equivalent site:*	*164*	*181*	*261*	*269*	*292*		
*Boleophthalmus pectinirostris*	Terrestrial mudskipper	LWS1	A	H	Y	T	A	553 nm	[28]
		LWS2	A	H	F	A	A	531 nm	
*Periophthalmus magnuspinnatus*	Terrestrial mudskipper	LWS1	S	H	Y	T	A	560 nm	
		LWS2	A	H	F	T	A	546 nm	
*Neogobius melanostomus*	Freshwater temperate rivers and lakes	LWS1	S	H	Y	T	A	560 nm	This study
		LWS2	S	H	F	T	A	550 nm*	This study

*Predicted by the key tuning sites, and Y261F shift of 10 nm; Yokoyama, 2008

Amino acid analysis of key tuning sites in Gobiidae red opsins proteins

**Fig. 3.**
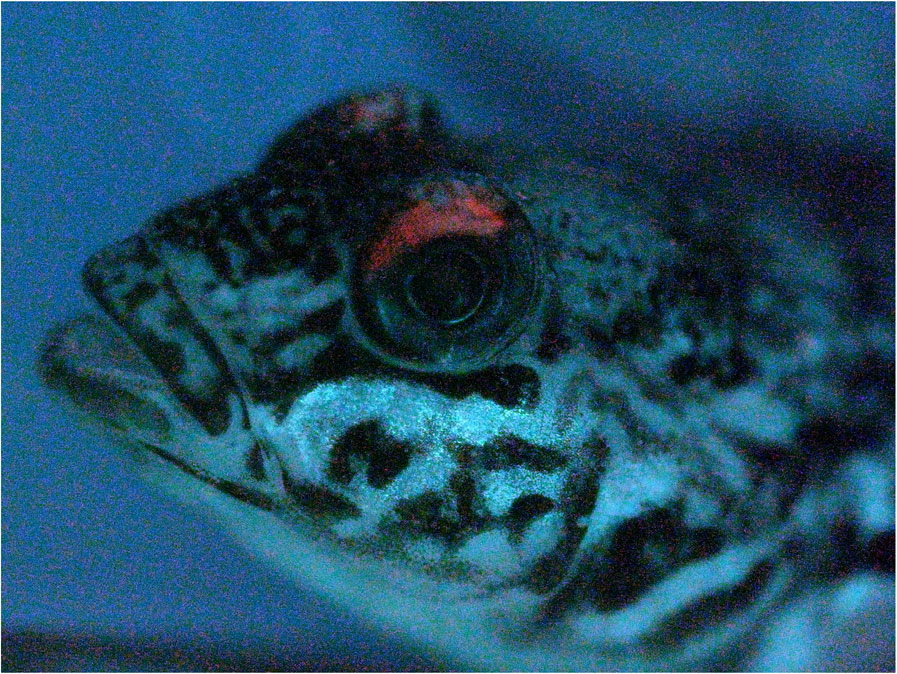
Red skin fluorescence in the round goby. Round gobies exhibit red fluorescence above the eyes when exposed to green light.

To further understand a potential ecological significance of the red opsin duplication, and since reef gobies are known to feature fascinating fluorescence patterns [69], we checked for the presence of red skin fluorescence in the round goby. Indeed, round goby individuals of both sexes and of all sizes (*n* = 10) feature weakly red fluorescent crescents above the eyes (Fig. 3). The macroscopic observation was confirmed with light sheet microscopy (preliminary data, not shown). Whether such pattern has any relevance for the putatively enhanced vision in the red spectrum remains elusive.

### Sensory perception genes: olfaction

Olfactory receptors (OR) in vertebrates are 7-transmembrane domain G-protein-coupled transmembrane proteins. They are expressed in neurons embedded in the olfactory lamellae. Mammals usually have several hundred OR genes (~ 400 in human (*Homo sapiens*), and ~ 1000 genes in mouse (*Mus musculus*)) that cluster in two major types [70]. Teleost fishes possess fewer OR genes but feature a higher diversity (5 kinds of type 2 ORs in teleosts as compared to 2 kinds of type 2 ORs in mammals) [71]. The binding properties of individual ORs, especially in fishes, are virtually unexplored.

**Fig. 4.**
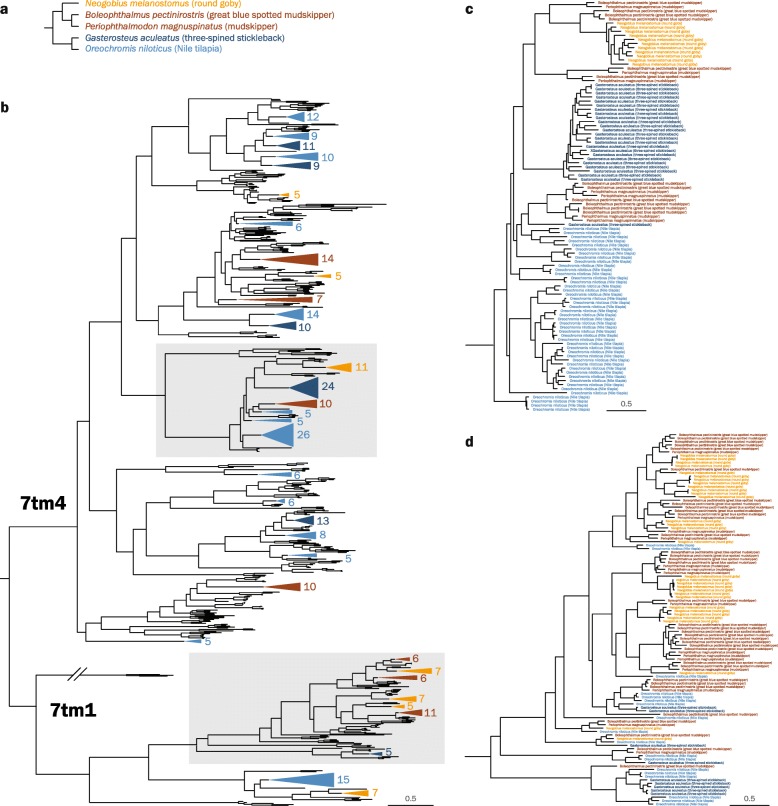
Phylogenetic tree of percomorph olfactory receptor protein sequences. **a** Phylogenetic relationship among five analyzed percomorph species, i.e., three gobiids (*Neogobius melanostomus*, *Boleophthalmus pectinirostris*, *Periophthalmodon magnuspinatus*), one cichlid (*Oreochromis niloticus*), and one stickleback (*Gasterosteus aculeatus*). **b** Maximum likelihood phylogenetic tree constructed with adrenergic receptors as outgroup. Sequences were identified de novo except for nile tilapia (*Oreochromis niloticus*; blue). Branches magnified in **c** and **d** are highlighted with gray boxes. An expanded view of the tree is available as Additional file 4: Figure S3. **c** Branch of the 7tm4 family featuring large independent expansions in all species analyzed. **d** Branch of the 7tm1 family featuring several expansions in Gobiidae (red, orange) that are not paralleled in other percomorph species (blue)

We identified 112 putative olfactory receptor genes in the round goby genome. To put this result into evolutionary context, all analyses were carried out in comparison with two Gobiidae species (blue-spotted mudskipper, *Boleophthalmus pectinirostris* and giant mudskipper, *Periophthalmodon magnuspinatus*) and two percomorph species (threespine stickleback, *Gasterosteus aculeatus* and nile tilapia, *Oreochromis niloticus*; Fig. 4a). The round goby presented a similar number of ORs (*n* = 112) to the giant mudskipper (*Periophthalmodon magnuspinatus*) (*n* = 106) and stickleback (*Gasterosteus aculeatus*) (*n* = 115), notably less than the blue-spotted mudskipper *(Boleophthalmus pectinirostris)* (*n* = 154) and near half the amount compared to nile tilapia (*Oreochromis niloticus*) (*n* = 214). We find that all ORs belong to one of two transmembrane domain subtypes according to the Pfam database (7tm4 or 7tm1; Fig. 4b; see Additional file 4: Figure S3 for expanded branches). This matches a previous large-scale phylogenetic analysis which identified two main types of olfactory receptor genes in vertebrates [71]. The functional differences between the domain subtypes are unclear, but their different consensus sequences may confer distinct biochemical properties.

Our analyses identify several cases of clade-specific gene expansions. Certain OR genes are expanded in parallel in several lineages (Fig. 4c). Likely, those expansion events are the result of clade-restricted gene duplications, although a secondary role for gene conversion after species divergence cannot be ruled out. While the nile tilapia (*Oreochromis niloticus*) features the greatest overall amount of expansions, the round goby presents the highest number of genes and expansions within the 7tm1 subfamily, a trend that is consistent in the other Gobiidae species (Fig. 4d).

### Response to the environment: detoxification

The CYP gene superfamily is an essential part of the defensome, a collection of genes that provide protection against harmful chemicals [38]. Vertebrate genomes contain between 50 and 100 CYP genes. The genomes of fugu (*Takifugu rubripes*), zebrafish (*Danio rerio*), and channel catfish (*Ictalurus punctatus*), for example, encode *n* = 54 [72], *n* = 94 [73], and *n* = 61 [74] CYP genes respectively. Expansions of individual CYP families occur in both mammals and fish. For example, zebrafish (*Danio rerio*) have three times as many CYP2 family members (*n* = 40) as most other vertebrate species (*n* = 13–15), and similar expansions of CYP2 genes have been observed in mice and rats [75].

We find that the round goby genome contains few CYP genes. We identify 25 complete or partial CYP genes, as well as 21 gene fragments. Pseudogenes are common for CYP genes [72, 73, 76], which is why strict annotation criteria are applied first before smaller fragments are considered. In total, the genome contains approximately 50 CYP genes (see Additional file 5: Table S2 for annotation details of genes, partial genes, and gene fragments).

**Fig. 5.**
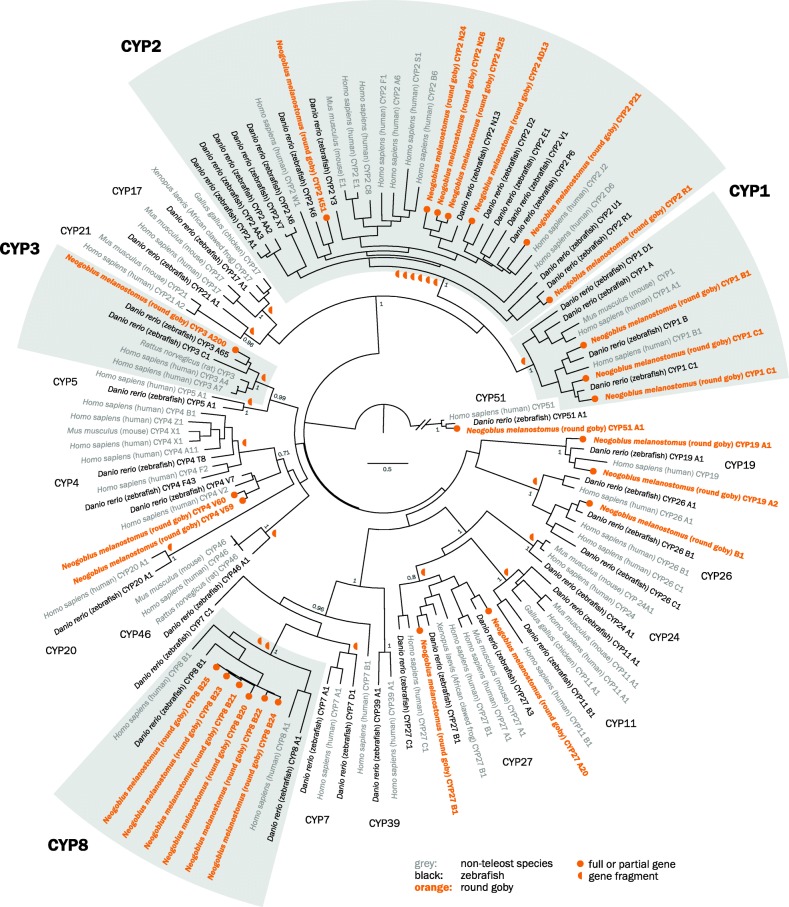
Phylogenetic tree of vertebrate CYP protein sequences. Maximum likelihood phylogenetic tree of round goby (*Neogobius melanostomus*), zebrafish (*Danio rerio*), human (*Homo sapiens*), chicken (*Gallus gallus*), frog (*Xenopus laevis*), mouse (*Mus musculus*), and rat (*Rattus norvegicus*), with 100 bootstraps, rooted with the CYP51 family. Detoxification genes CYP1–3 do not feature expansions, while a family with largely unknown function, CYP8, is expanded to six members (see gray boxes). Non-fish vertebrates are indicated in gray. Gene fragments too short for tree building but attributable to a certain family are indicated by orange half circles next to the root of the respective family

When including gene fragments, all expected CYP families are present in the round goby, and the phylogenetic analyses show the expected relationships between gene families and between vertebrates (Fig. 5). Fish and most vertebrates have CYP genes from 17 families (CYP 1–5, 7, 8, 11, 17, 19, 20, 21, 24, 26, 27, 46 and 51) [72], while the CYP39 family occurs in humans (*Homo sapiens*) and zebrafish (*Danio rerio*), but not in fugu (*Takifugu rubripes*) or channel catfish (*Ictalurus punctatus*) [72–74]. In the round goby, the complete or partial genes could be assigned to 9 CYP families (CYP 1–4, 8, 19, 26, 27 and 51). The families CYP7, CYP11, CYP17, and CYP21 were present among the sequence fragments.

CYP1, 2, 3 and to a lesser extent CYP4 proteins are responsible for the oxidative metabolism of xenobiotic compounds (pollutants, drugs, etc.). In rodents and humans, the CYP1 family metabolizes planar cyclic aromatic hydrocarbon compounds (reviewed in [77]), the CYP2 family metabolizes structurally diverse drugs, steroids, and carcinogens, the CYP4 family catalyzes the ω-hydroxylation of the terminal carbon of fatty acids and xenobiotics, and CYP3 genes metabolize a range of structurally different compounds in the liver and intestines. Over 50% of all pharmaceutical compounds are metabolized by CYP3A genes in human. Contrary to expectations derived from the occurrence of round goby in contaminated sites, the classical detoxification families CYP1–3 were not expanded (Fig. 5). The goby genome contains three or four CYP1 genes: one CYP1B gene, two CYP1C genes, and one CYP1A fragment. The latter lacks two main characteristics (I- and K-helix) and could therefore be a pseudogene. As expected for a vertebrate [75], the genome contains many CYP2 genes. The most important fish CYP2 families were represented, including CYP2J, CYP2N, CYP2Y, and CYP2AD. Finally, the round goby had a single CYP3A gene and a potential CYP3A fragment. This is somewhat unusual because fish often feature species-specific CYP3 subfamilies in addition to CYP3A. For example, medaka also contains CYP3B genes, zebrafish (*Danio rerio*) CYP3C genes, and Acanthopterygii fish CYP3D genes [78].

In addition, we find that the round goby genome contains six CYP8 genes, which is more than expected based on observations from the other gobies. The closely related blue-spotted mudskipper (*Boleophthalmus pectinirostris*) has only two CYP8 genes (XM_020924471 and XM_020919000.1; about 73–85% identity); no sequences were found in other mudskipper species. Accordingly, we assume that the CYP8B genes have undergone species-specific tandem duplications in the round goby, as is also known for the subfamilies CYP2AA, CYP2X, and CYP2K in zebrafish (*Danio rerio*) [75]. Five round goby CYP8 genes locate to the same contig with high sequence similarity (~ 90%), which is similar to zebrafish (*Danio rerio*) CYP8B1–3 that also colocalize on the same chromosome [73]. Misidentification of closely related CYP7 and CYP39 genes as CYP8 is unlikely given the colocalization and high sequence similarity. The function of the expansion is presently unclear, although expression patterns in zebrafish (*Danio rerio*) suggest a role in the early embryo [73]. In humans, CYP8 genes act as prostacyclin synthases that mediate steroid metabolic pathways in bile acid production or prostaglandin synthesis [79]. Based on structural similarities with yeast proteins, CYP8 genes might also have E3 ubiquitin ligase activity. The almost identical crystal structures of zebrafish (*Danio rerio*) and human CYP8A1 suggest similar functions in fish and mammals [80].

### Response to the environment: osmoregulation

Osmotic homeostasis depends on passive ion and water uptake through cell membranes and the intercellular space, on the active uptake or excretion of ions, and on the production and accumulation of osmolytes. To understand the ability of round goby to colonize a wide range of salinities, we annotated the round goby repertoire of osmoregulatory genes and compared it to that of a stenohaline freshwater species (zebrafish; *Danio rerio*) and of distant as well as related euryhaline species (nile tilapia, *Oreochromis niloticus*; blue-spotted mudskipper, *Boleophthalmus pectinirostris;* and threespine stickleback, *Gasterosteus aculeatus*).

**Fig. 6.**
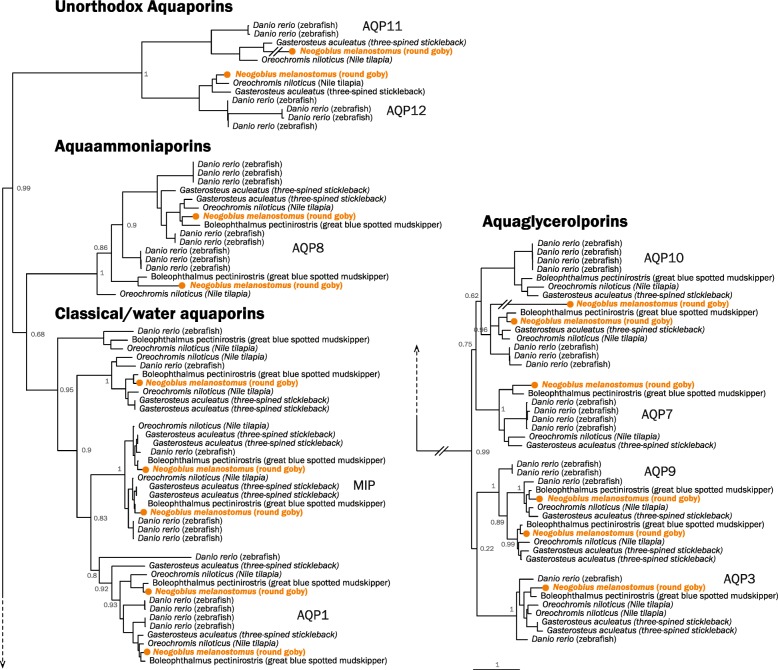
Phylogenetic tree of fish aquaporin proteins. Maximum likelihood tree with 100 bootstraps of round goby (*Neogobius melanostomus*, orange) in relation to cyprinid zebrafish (*Danio rerio*) and percomorph threespine stickleback (*Gasterosteus aculeatus*), nile tilapia (*Oreochromis niloticus*), and blue-spotted mudskipper (*Boleophthalmus pectinirostris*). The main classes of aquaporins are labeled with human genes names

Passive ion and water transport across membranes (transcellular permeability) depends on the superfamily of aquaporin proteins. Aquaporins transport water (classical aquaporins), water and glycerol (aquaglyceroporins), ammonia (aquaammoniaporins), or additional undescribed molecules (unorthodox aquaporins). Primary sequences are only moderately conserved between the classes (approximately 30% identity), but all aquaporins share six membrane-spanning segments and five connecting loops. We find 15 aquaporin genes in the round goby, which compares to the number in human (*Homo sapiens*) (*n* = 13) or zebrafish (*Danio rerio*) (*n* = 20) and is lower than in the euryhaline Atlantic salmon (*n* = 42) [81, 82]. With 5 classical water aquaporins, 6 aquaglyceroporins, 2 aquaammonioporins, and 2 unorthodox aquaporins, the round goby features the same types of aquaporins as freshwater stenohaline fish (e.g., zebrafish, *Danio rerio*) and highly euryhaline fish (e.g., tilapia, *Oreochromis niloticus*; Fig. 6).

Ion and water flow between cells in epithelia (paracellular permeability) is regulated by tight junctions, of which claudin and occludin proteins are the most important components. Mammalian genomes contain ~ 20 claudin genes, invertebrates such as the nematode *Caenorhabditis elegans* or the fruit fly *Drosophila melanogaster* contain 4–5 genes, and fish often feature large expansions. For example, the fugu (*Takifugu rubripes*) genome contains 56 claudins, of which some occur in clusters of > 10 genes [83]. The round goby genome features 40 claudin paralogues, which is in line with numbers known from other fish. All human (*Homo sapiens*) claudin genes were represented as homologs (see Additional file 6: Figure S4 for a phylogenetic tree of claudins), and the round goby genome contains one occludin gene in each of the two known subclades of the protein family (see Additional file 7: Figure S5 for a phylogenetic tree of occludins).

**Fig. 7.**
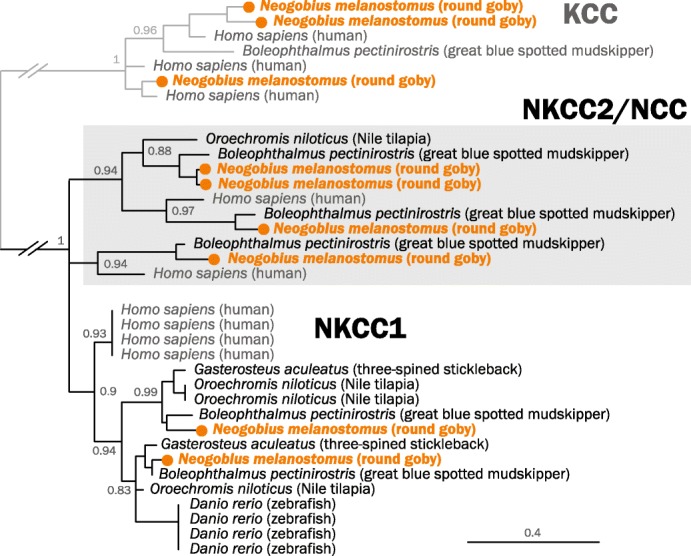
Phylogenetic tree of human and fish sodium/potassium/chloride co-transporter proteins (NKCC). Maximum likelihood tree with 100 bootstraps of round goby (*Neogobius melanostomus*, orange), zebrafish (*Danio rerio*), threespine stickleback (*Gasterosteus aculeatus*), nile tilapia (*Oreochromis niloticus*), blue-spotted mudskipper (*Boleophthalmus pectinirostris*), and as non-fish representative human (*Homo sapiens*, gray). Gobiidae feature more NKCC2 genes (gray box). Potassium/chloride co-transporters (KCC) indicated in gray type and with gray lines on top are used as outgroup

In the kidney, intestine, and gills, fish use active ion transport (mostly sodium transporters) to maintain osmotic balance. Mechanisms mediating sodium uptake include electroneutral Na+/H+ exchange via the NHE3b protein, Na+/Cl− co-transport via the NCC protein, and coupling of Na+ absorption with H+ secretion by a V-type H+-ATPase [84]. We find 12 Na+/H+ exchanger genes, 5 Na+-K+-ATPase catalytic alpha subunits, and 6 Na+-K+-ATPase regulatory beta subunits in the round goby genome. The round goby thus contains the same types of genes, but less copies, than either zebrafish (*Danio rerio*) or nile tilapia (*Oreochromis niloticus*) (see Additional file 8: Figure S6 for phylogenetic trees of Na+/H+ exchangers and Na+-K+-ATPases). We find that round goby, and also mudskippers, feature an interesting distribution of Na+/Cl- co-transporters to subgroups; while most zebrafish (*Danio rerio*) and nile tilapia (*Oreochromis niloticus*) Na+/Cl- co-transporters belong to the NKCC1 subgroup, Gobiidae feature more genes in the NKCC2 subgroup (Fig. 7).

Finally, fish produce osmolytes to actively take up and retain water. In particular, the cyclic polyol myo-inositol is used by euryhaline teleosts to acclimate to high salinity. Two enzymes are required for its production: myo-D inositol 3-phosphate synthase (MIPS) and inositol monophosphatase (IMPA). In addition, some fish actively accumulate myo-inositol with a sodium/myo-inositol co-transporter (SMIT) [85, 86]. This transporter is of particular importance for marine fish exposed to high salt concentrations [87, 88], while freshwater fish lack a SMIT gene (e.g., the freshwater stenohaline zebrafish (*Danio rerio*) lacks the SMIT gene). The presence of SMIT has therefore been proposed to be a critical prerequisite for high salinity tolerance in fish [89]. We find that the round goby genome contains MIPS and IMPA, and importantly, also a SMIT gene (see Additional file 9: Figure S7 for phylogenetic trees of MIPS, IMPA, and SMIT genes).

### Response to the environment: immune system

It has been speculated that invasion success may relate to the ability to fight novel immune challenges [41]. We therefore characterized key genes related to the immune system, focusing on genes that span both the innate and adaptive immune system such as pattern recognition receptors, selected cytokines and chemokines, antigen presentation, T cell surface receptors, and antibodies (see Additional file 10: Table S3 for a list of genes analyzed, and Additional file 11: Table S4 for a list of queries used against the round goby genome).

We find that the round goby genome features a classical adaptive immunity setup (Table 3). Vertebrate adaptive immunity is characterized by the Major Histocompatibility Complex (MHC) class I and MHC class II proteins and their regulators. MHCI presents antigens derived from a cell’s intracellular environment, while MHCII presents antigens derived from material engulfed by macrophages, B cells, or dendritic cells [90]. We find 26 full-length MHCI sequences from the classic U-lineage and one sequence from the teleost-specific Z-lineage [91] (see Additional file 12: Table S5 for MHCI annotation details). MHCII is represented by 8 alpha (2 fragments) and 9 beta copies (see Additional file 13: Table S6 for MHCII annotation details). The uneven numbers may be attributed to assembly issues, but also additional small fragments were not further investigated (data not shown). We also identify the key MHC-supporting peptides Beta-2-Microglobulin, *CD74*, *TAP1/2* and *tapasin*. Beta-2-Microglobulin (*B2M*) is present in two copies, one of which contains several indels, a diverged region, and no stop codon and thus may be a pseudogene. The round goby has two copies of *TAP2*, which promotes the delivery of peptides to MHCI (annotated as *TAP2* and *TAP2T;* see Additional file 14: Figure S8 for a phylogenetic tree of TAP genes). Two *TAP2* genes have also been described in zebrafish (*Danio rerio*), and our results thus suggest this is a conserved feature among teleosts [92]. In addition, we identify the MHC transcriptional regulators *CIITA* and *NLRC5*. The presence of the thymus transcription factor *AIRE* and the T cell receptors *CD4* and *CD8* confirms the presence of helper T cells and cytotoxic T cells in the round goby (see Additional file 15: Table S7 for annotation details of adaptive immunity genes).

**Table 3:** Adaptive immunity

Gene	NEME annotation	Contig annotation	Start	End	Strand
CIITA	NEME_493	Contig_2585	3,985,719	3,993,128	Antisense
AICDA	NEME_58	Contig_447	597,424	599,014	Sense
AIRE	NEME_9	Contig_79	14,106,230	14,113,573	Antisense
B2M	NEME_421	Contig_2242	363,050	363,352	Antisense
B2M_pseudo	NEME_421	Contig_2242	368,352	368,721	Antisense
CD4	NEME_213	Contig_1334	340,445	348,248	Sense
CD74	NEME_71	Contig_593	791,743	796,652	Antisense
CD8a	NEME_729	Contig_3231	634,222	648,487	Antisense
CD8b	NEME_729	Contig_3231	656,030	660,462	Antisense
RAG1	NEME_106	Contig_787	4,690,414	4,695,142	Sense
RAG2	NEME_106	Contig_787	4,699,042	4,700,651	Antisense
TAP1	NEME_582	Contig_2864	694,776	722,339	Sense
TAP2	NEME_387	Contig_2107	2,987,106	2,993,287	Antisense
TAP2T	NEME_299	Contig_1786	3,697,645	3,704,089	Sense
Tapasin	NEME_387	Contig_2107	3,111,989	3,119,308	Sense

Overview of manually annotated key adaptive immune genes

Similarly, the humoral adaptive immune response (also termed the B cell-mediated immune response) is intact in the round goby. Humoral immunity in fish is characterized by three antibody isotypes consisting of immunoglobulin heavy chains delta (IgD), mu (IgM), and tau (IgT). We identify a contig-spanning immunoglobulin heavy chain locus (see Additional file 16: Figure S9 for a graphic representation of the immunoglobulin locus) containing 8 delta constant domains, and 4 constant mu domains, as well as genes responsible for heavy chain recombination and immunoglobulin hypermutation (*RAG1/2* and *AID(AICDA)*; Table 3). There is no evidence for the presence of immunoglobulin tau constant domains, which are commonly found in carps and salmonids [93].

**Fig. 8.**
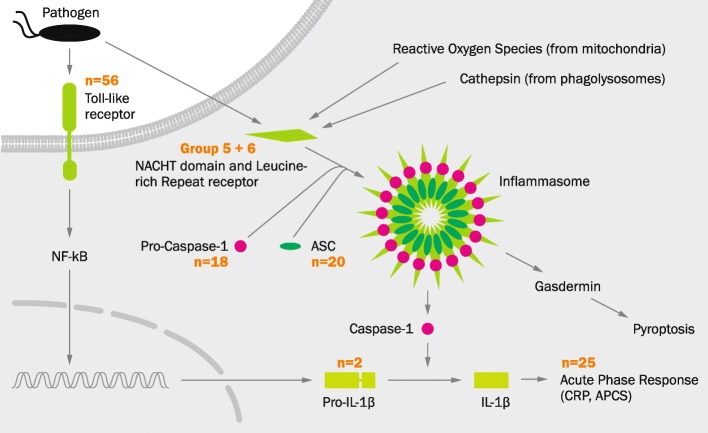
The inflammasome pathway. Pathogen-associated patterns are recognized by pattern recognition receptors such as Toll-like receptors at the cell surface (TLRs), or NACHT domain and Leucine-rich Repeat containing receptor (NLRs) in the cytoplasm. This interaction triggers the transcription of cytokine precursors via NFkB, and the activation and assembly of inflammasome components (NLRs, Pro-Caspase-1, and ASC). Inflammasome-activated Caspase 1 then initiates the maturation of cytokines and an acute phase inflammatory response (CRP, APCS proteins), and/or pyroptosis through gasdermin. Several components of the pathway are expanded in the round goby (gene numbers in round goby, or novel groups for NLRs, are indicated in orange)

While round goby adaptive immunity conforms to vertebrate standards, its innate immune repertoire displays remarkable and unusual features. We find that all components of the inflammasome (a signaling pathway involved in inflammatory responses; Fig. 8) are expanded. Inflammasome assembly is activated through pathogen pattern recognition receptors [94], and ultimately triggers a local or systemic acute phase response by producing IL-1 family cytokines [94, 95] and/or promotes cell death via pyroptosis [95]. In the round goby genome, components of the entire cascade (pattern recognition receptors, ASC adaptor proteins, IL-1, and acute phase proteins) are present in unexpectedly large numbers (Fig. 8; see Additional file 17: Table S8 for detailed annotation data). In the following, our findings are described step-by-step from the cell surface down to the acute phase response.

**Fig. 9.**
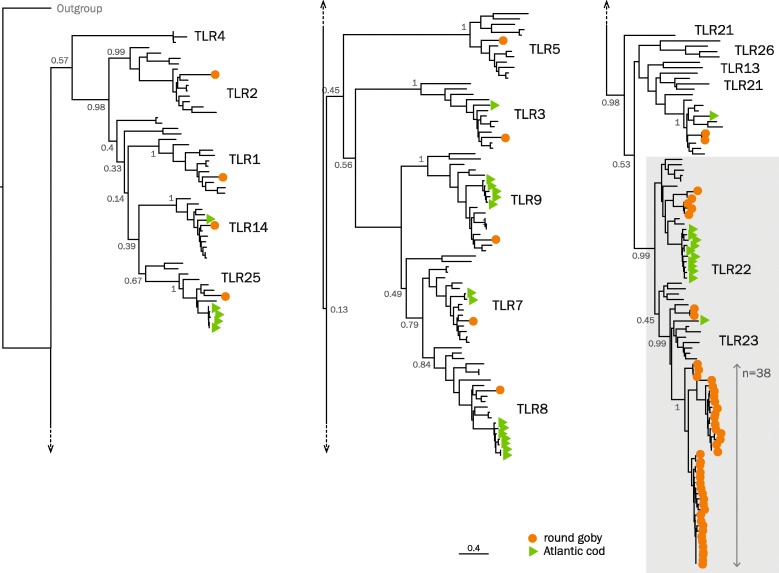
Phylogenetic tree of teleost Toll-like receptor protein sequences. A maximum likelihood phylogenetic tree run with the JTT substitution model and 500 bootstrap replicates on the transmembrane, linker, and TIR domain of all TLRs found in a selected set of teleosts in the Ensembl database, the Atlantic cod genome version 2, and all manually investigated Gobiiformes. A TLR sequence from the lancelet *Branchiostoma belcheri* was used as an outgroup and the root was placed upon its corresponding branch. Green triangles, Atlantic cod. Orange circles, round goby. Gray box, TLR22 and TLR23

Perhaps the best studied pattern recognition receptors are the Toll-like receptors (TLRs), pathogen-recognizing molecules that are generally expressed either at the plasma membrane or on the endosomal membranes. Currently, approximately 20 types of TLR genes distributed across 6 subfamilies are phylogenetically supported in vertebrates. Most vertebrate genomes contain one to three members of each subfamily, with occasional species-specific small gene expansions. Examples are 4 copies of TLR14 in frog (*Xenopus laevis*), 3 copies of TLR5 in channel catfish (*Ictalurus punctatus*), or 2 copies of TLR2 in chicken (*Gallus gallus*) [96–99]. As expected for a teleost, the round goby genome does not contain the LPS-detecting TLR4 genes. However, in total, we find 56 TLRs, of which 40 appear to originate from an expansion of Toll-Like Receptor 23-like genes (Fig. 9). In other *Gobiidae*, we find that *TLR22* and *TLR23* are at most moderately expanded to 6–13 copies (see Additional file 18: Figure S10 for a phylogenetic tree of *Gobiidae* TLRs). The extent of the expansion of *TLR23* in round goby exceeds what is observed for *TLR22* in Atlantic cod and overlaps with the extreme estimates (estimates due to low coverage of underlying genome data) of *TLR23* in selected codfishes as well as European perch (*n* = 17), kissing gourami (*n* = 14), and glacier lantern fish (*n* = 49) [99]. Phylogenetically, the identified TLR23 sequences form three clades, of which two are specific to *Gobiidae*, while the third also contains TLR23 sequences from other teleosts (Additional file 18: Figure S10). In terms of genomic location, round goby TLRs 22 and 23 were distributed across several contigs with some copies arranged in tandem, which suggests several independent duplication events.

**Fig. 10.**
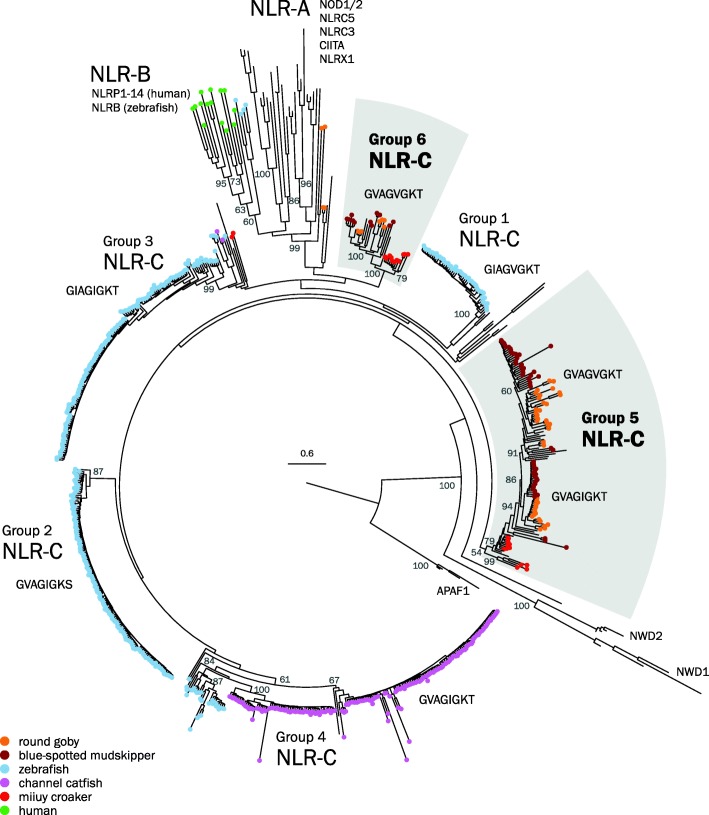
Phylogenetic tree of the NACHT domain and Leucine-rich Repeat containing receptor (NLR) nucleotide-binding domain sequences in round goby, blue-spotted mudskipper (*Boleophthalmus pectinirostris*), zebrafish (*Danio rerio*), (*Ictalurus punctatus*), miiuy croaker (*Miichthys miiuy*), and human (*Homo sapiens*). Maximum Likelihood phylogenetic tree with 500 bootstraps rooted at the split between NB-ARC (found in APAF) and NACHT domains (present in all the other NLRs). NB-ARC domains from APAF1 orthologs were used as an outgroup. Bootstrap values are shown for nodes that determine an entire cluster. The tree resolves all three major classes of vertebrate NLRs (NLR-A, NLR-B, NLR-C). NLR-A genes were conserved in all analyzed species; no NLR-B genes were found in the gobies. Six groups of NLR-C genes were identified, four of which are exclusive to zebrafish (*Danio rerio*) (groups 1–4) and two contain only sequences from gobies (groups 5 and 6, gray boxes and bold print). Lineage-specific expansions are displayed with colored endpoints. Within the goby-specific groups, lineage-specific expansions can be seen for both round goby (orange) and blue-spotted mudskipper (*Boleophthalmus pectinirostris*) (brown). The placement of sparse miiuy croaker genes in group 3 and round goby genes in NLR-A clusters is not well supported and presumably an artifact. The characteristic Walker A motifs are shown next to each subgroup, with group 5 featuring 2 different motifs

For intracellular pathogen recognition receptors of the NACHT domain and Leucine-rich Repeat containing receptor (NLR) family, we identify two new, previously undescribed families (groups 5 and 6) present in the round goby and also in the blue-spotted mudskipper (*Boleophthalmus pectinirostris*) (Fig. 10). These NLR subtypes are also present in the miiuyi croaker (*Miichthys miiuy*), but not in otocephalid fish such as zebrafish (*Danio rerio*) and channel catfish (*Ictalurus punctatus*) (Fig. 10).

NLRs have diverse roles from direct pathogen recognition to transcriptional regulation of the MHC (NLRs CIITA and NLRC5) and contribute to inflammasome activation [100]. Mammalian genomes display 20–40 NLRs in families NLR-A and NLR-B, while fish also feature a fish-specific subfamily (NLR-C) [101] and a much expanded NLR repertoire (e.g., 405 NLR-C genes in zebrafish, *Danio rerio*) [102, 103]. Other species in which the NLR-C genes have been extensively investigated include the channel catfish (*Ictalurus punctatus*) and miiuy croaker (*Miichthys miiuy*) [104–106].

The round goby genome contains at least 353 NLRs (see Additional file 17: Table S8 for annotation details), which include 9 highly conserved vertebrate NLRs (*NOD1*, *NOD2*, *NLRC3*, *NLRC5*, *NLRX1*, *NWD1*, *NWD2*, *APAF1*, *CIITA*) as well as 344 NLR-C genes. Fish NLRs cluster into 6 groups of which 2 represent novel NLR-C clades (groups 5 and 6, Fig. 10). The novel groups are supported by phylogenetic analyses as well as motif presence/absence (Table 4). NLR-C groups are characterized by highly conserved versions of the sequence motif Walker A. The most common sequence for Walker A observed in both goby NLR-C groups, GVAGVGKT, is not associated with any of the four major NLR-C groups in zebrafish (*Danio rerio*) [102]. Also, NLR subtypes often carry group-specific combinations of the protein-protein interaction domain PYD and/or B30.2 domain. This holds true for *Gobiidae* NLR-C groups, since only group 5 NLRs can carry an N-terminal PYD domain and/or a C-terminal B30.2 domain [102], similar to the zebrafish (*Danio rerio*) group 1 and 2 NLRs (Table 4). In contrast, some group 6 NLRs have C-terminal CARD domains, which in both human (*Homo sapiens*) and zebrafish (*Danio rerio*) are attached to specific inflammasome-associated NLR-B genes [107]. The round goby C-terminal CARD-containing NLRs are found on the same few scaffolds and share a high degree of sequence similarity, indicative of a recent expansion. This expansion is absent from blue-spotted mudskipper (*Boleophthalmus pectinirostris*) and thus restricted to the round goby lineage. Many other group 6 NLRs are fragmented, with large insertions in the middle of their conserved 2-kb exon, indicative of either pseudogenization or the acquisition of an intron.

**Table 4:** NLR-C characteristics

Group	Identified in this study	Walker A	Last residues of the largest exon	PYD?	B30.2?
1		GIAGVGKT	L(I/M)PVVKNT(T/R)RA	+	+
2		GVAGIGKS	LSAVIKTSKRA	+	+
3		GIAGIGKT	L(IP/TA)AV(R/S)NC(RK/TR/RR)A	−	+
4		GVAGIGKT	LPV(I/V)xxxx(A/V)x	−	−
5	x	GVAG(V/I)GKT	(L/M)PV(V/I)KASxK(A/V)	+	+
6	x	GVAGVGKT	L(I/V)P(A/V)VRNCRKA	−	−

Key features of each of the six NLR-C subgroups as identified from zebrafish (*Danio rerio*) and round goby. Each of the groups is characterized by the presence of a highly conserved Walker A Motif and either the presence or absence of specific C- and N-terminal domains (PYD and B30.2). x denotes a variable amino acid, + and − denote whether the denoted domains are characteristically present/absent in the group

With the same approach, we identified ~ 50 NLRs in miiuy croaker (*Miichthys miiuy*), which corresponds to the number reported previously [105], and ~ 160 NLR-C genes in the latest channel catfish (*Ictalurus punctatus*) assembly (ASM400665v2, sequenced with 57.7x PacBio reads). Our analyses confirm earlier observations [106] that many of the channel catfish (*Ictalurus punctatus*) NLRs appear to be pseudogenes, with stop codons interrupting the coding sequence of the 2-kb exon. Channel catfish (*Ictalurus punctatus*) NLRs fall into the same four groups as in zebrafish (*Danio rerio*), with most channel catfish (*Ictalurus punctatus*) NLR-C genes in groups 2 and 4 (Fig. 10), and only few in groups 1 and 3. In contrast, the miiuy croaker (*Miichthys miiuy*) NLR genes cluster with the novel NLR-C subtypes identified in gobies (groups 5 and 6).

**Fig. 11.**
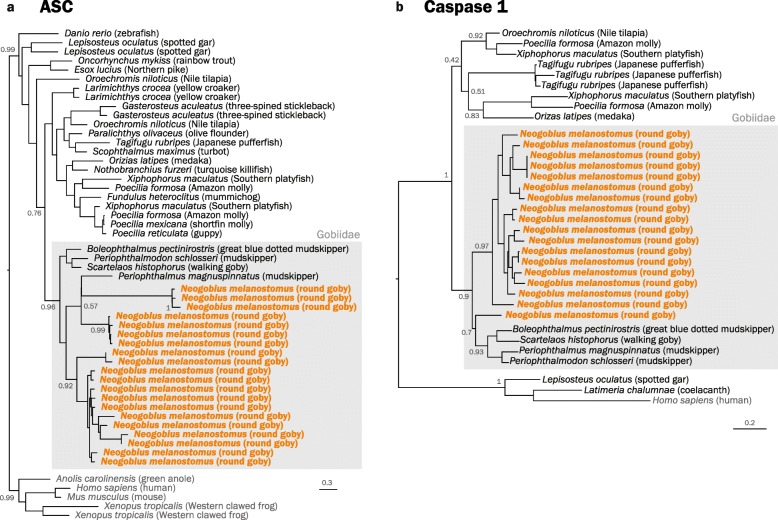
**a** Phylogenetic tree of gnathostome ASC protein sequences. Maximum Likelihood phylogenetic tree with 500 bootstraps rooted at the split between tetrapods and ray-finned fish. Tetrapods were used as outgroup. Round goby is indicated in orange. Gobiidae are highlighted with a gray box. The goby sequences form a clear separate cluster, with a large expansion apparent in the round goby. **b** Phylogenetic tree of gnathostome Caspase 1 protein sequences The Caspase 1 tree comprises all protein sequences annotated as CASP1 in the investigated Gobiidae genomes aligned together with reference sequences from Ensembl and GenBank. The root was placed on the branch containing the mammalian sequences

Once activated, some NLRs (including those with a C-terminal CARD) can oligomerize and form a structure termed “inflammasome” in order to activate specific caspases (usually Caspase 1; Fig. 8). The interaction between NLRs and the caspase are mediated by the adaptor protein ASC (also known as PYCARD), which itself oligomerizes into large structures known as “specks” [108]. Vertebrates have 1–2 copies of ASC, which are characterized by a distinct combination of a single PYD and CARD domain. In the round goby genome, we find 20 cases of this domain combination. Since the genomes of other gobies contain 1–2 PYD-ASC combinations, the expansion appears to be specific to the round goby (Fig. 11a). The effector protein Caspase 1 is present as one gene in humans (*Homo sapiens*) and as two genes in zebrafish (*Danio rerio*). We find that the round goby genome features an expansion to 18 copies. Interestingly, some of those genes appear to contain a CARD domain (as seen in mammals and several species of fish) while others have PYD (as seen in zebrafish *Danio rerio*). The occurrence of both types in a single species suggests that a caspase with both domains may have existed in the common ancestor of fish and tetrapods, with most lineages having retained only one of the two and round goby retaining both. However, since round goby Caspase 1 genes are the result of a single expansion event specific to this species (Fig. 11b), a recurrent re-acquisition of PYD is a valid alternative scenario. In addition to Caspase 1 genes, Caspase 3 (a key component of apoptosis which may be activated by Caspase 1) is also expanded to 5 copies. Caspase 4 and 5, on the other hand, appear to be absent.

Finally, we find that genes encoding for two peptides produced in the course of inflammation, the acute phase reactants C-reactive protein (CRP) and serum amyloid component P (APCS), are expanded to a total of 25 copies (compared to < 2–7 in fish, and 5–19 in the other *Gobiidae*). In fish, CRP and APCS are closely related and cannot be distinguished based on BLAST scores or phylogeny. As seen in other fish species, all investigated CRP/APCS sequences resolve into two major phylogenetic clades, with the mammalian sequences in a third (see Additional file 19: Figure S11 for a phylogenetic tree of CRP/APCS).

### Adaptation to novel environments: epigenetic regulators

**Fig. 12.**
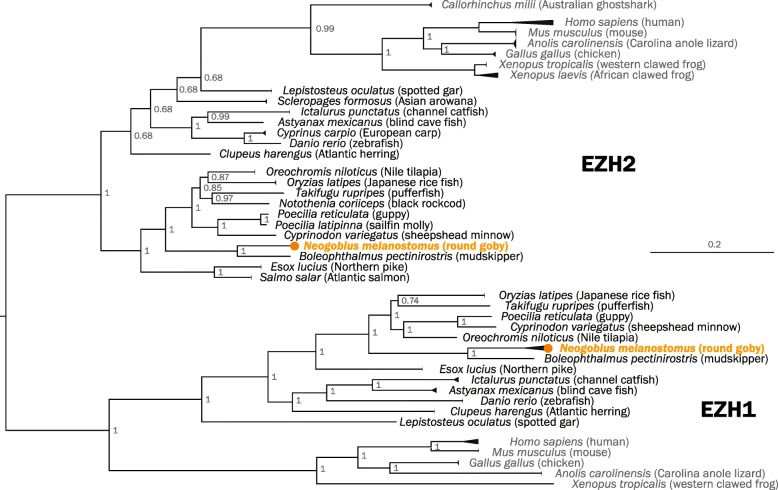
Phylogenetic tree of vertebrate EZH proteins. Midpoint-rooted Bayesian phylogenetic tree. The Australian ghost shark (potential outgroup) is positioned within the poorly supported EZH2 branch. When rooting with Australian ghost shark, teleost EZH2 genes cluster with EZH1 (data not shown). Round goby is indicated in orange

The PRC2 complex establishes and maintains gene repression [109] and thus represents a plasticity-restricting mechanism. The complex mediates di- and trimethylation of lysine 27 on histone H3 and contains four proteins: a catalytic subunit (either *enhancer of zeste* EZH1 or EZH2), *suppressor of zeste* SUZ12, *embryonic ectoderm development* EED, and *RB Binding Protein 4* RBBP4 [54]. In mammals, the alternative catalytic subunits EZH1 and EZH2 have partially complementary roles [110, 111], and requirements for the two alternative catalytic subunits differ between species—in contrast to mammals, zebrafish (*Danio rerio*) develop in the absence of either catalytic subunit [112, 113]. We find that the round goby genome contains the usual complement of PRC2 components: two copies of SUZ12 (of which one appears quite diverged), one copy of EED, one copy of RBBP4, and two copies of EZH (with multiple isoforms determined by RACE experiments). For SUZ12, EED, and RBBP4, sequence-based identification was straightforward, and phylogenetic analyses followed the known phylogenetic relationships of fish, mammals, and other vertebrates (see Additional file 20: Figure S12 for phylogenetic trees of SUZ12, EED, and RBBP4). The catalytically active subunits EZH1 and EZH2 cluster with the closest species in the phylogeny, the blue-spotted mudskipper *(Boleophthalmus pectinirostris)* (Fig. 12), but the deeper relationships within EZH2 are poorly supported and may suggest a complex evolutionary history.

**Fig. 13.**
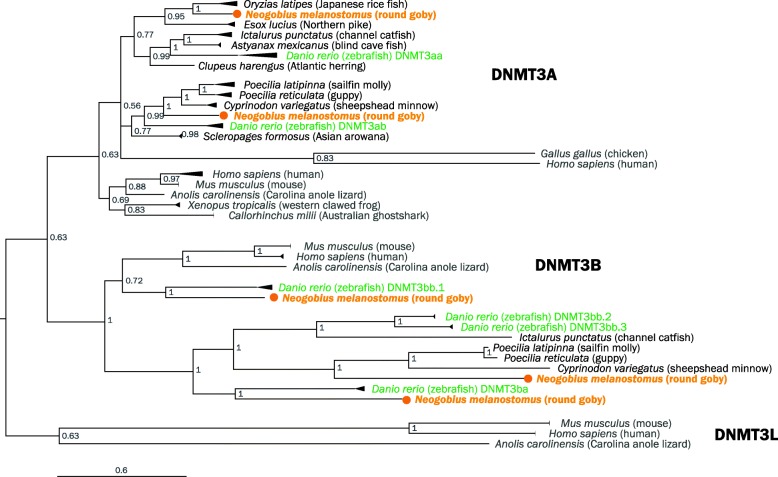
Phylogenetic tree of vertebrate DNMT3 proteins. Midpoint-rooted Bayesian phylogenetic tree. The Australian ghost shark (potential outgroup) is positioned among DNMT3A genes. Round goby is indicated in orange. Zebrafish (*Danio rerio*), the only other fish with well-annotated DNMT3 genes, is indicated in green

Methylation marks similarly regulate gene expression and are deposited by conserved enzymes called DNA methyltransferases (DNMTs). Mammals feature two types of DNMTs, DNMT3 (three genes A, B, and L), and DNMT1 (one gene) performing de novo and maintenance methylation, respectively, in a dynamic division of labor [114]. Interestingly, fish feature a variable repertoire of DNMT3 genes. Medaka (*Oryzias latipes*), fugu (*Takifugu rubripes*), zebrafish (*Danio rerio*), and common carp (*Cyprinus carpio*) have three, five, six, and 12 DNMT3 genes, respectively [115]. We find that the round goby genome features one DNMT1 that follows the expected phylogenies (data not shown), and five DNMT3 genes, of which two cluster with vertebrate DNMT3A sequences, and three with vertebrate DNMT3B sequences (Fig. 13). The number of DNMT3 genes in round goby corresponds to that seen in threespine stickleback (*Gasterosteus aculeatus*), fugu (*Takifugu rubripes*), and nile tilapia (*Oreochromis niloticus*) [116]. In general, the DNMT3 phylogeny is not well supported, which limits conclusions about the evolution of specific DNMT3 genes.

## Discussion

### General observations

Our analyses depict a genome that, in many respects, is similar to other teleost genomes. There is no evidence for recent genome duplications, and genome size, gene content, and GC content are within the ordinary range. Transposable elements can create genetic variation and have been proposed to support invasiveness [117], but repeat analyses do not reveal unusual transposon activities in the round goby. Small genome size has been proposed to foster invasiveness [118], but the round goby genome is not particularly small. Phylogenetic analyses reveal that many of the analyzed gene families conform to expectations. For example, green opsin gene duplications and the loss of the UV opsin are observed in many fish lineages [23]. Similarly, the expected gene families and overall gene complements are found for olfactory receptors, cytochrome P450, and osmoregulatory proteins, for adaptive immunity and epigenetic regulators. Multilocus sex determination has previously been suggested for many fish, including the goby *Ctenogobius shufeldti* [119], and indeed our data suggest a multigenic and/or environmental sex determination system is more likely than a large sex-determining region or a sex chromosome. Overall, these findings support the validity of the sequencing and assembly approach, and suggest that selected findings of interest are not artifacts. In addition, the round goby genome sequence also reveals several novel and interesting findings of which some pertain to teleost genomes in general, some to *Gobiidae*, and some to specific gene families, some of which warrant functional follow-up studies with regard to invasive potential.

Regarding annotation, our analyses reveal that some degree of care is warranted regarding gene models. De novo annotation without transcriptome data tends to be biased towards known and conserved genes, homopolymer sequencing errors may cause annotation errors, and fish proteins have diverged faster than mammalian homologs [120]. For example, 25% of human (*Homo sapiens*) genes cannot be identified in the pufferfish (*Takifugu rubripes*) [9]. Even in the well-characterized zebrafish (*Danio rerio*), targeted approaches have the potential to reveal additional novel genes [121]. BUSCO analyses show that most marker genes are covered as single copies indicating that the assembly process removed haplotypes in the assembly. The rate of recovered BUSCO groups indicate that some parts of the genome are not represented in the current annotation, either because they are absent from the assembly or because they have been masked. Some genes are fragmented (partial predictions) which may be due to the lack of transcriptome evidence but may also indicate that the assembly contains indels. We therefore encourage researchers to consider genome-wide blast searches in addition to a consultation of round goby gene models, and to account for small indels when annotating reading frames. We hope that extensive RNA sequencing data can be generated in the future to improve the predictions.

### Environmental perception

We find that the visual system of *Gobiidae* may be more efficient in the red parts of the light spectrum thanks to the presence of two potentially differently tuned LWS genes. This is intriguing considering the benthic lifestyle of gobies and their occurrence in turbid areas. In clear waters, red light from the sun is the least abundant part of the spectrum (and virtually absent below 15 m of depth) because red light penetrates least through water, but many organisms convert the deeply penetrating green and blue wavelengths into red. Indeed, the eyes of gammarids, a common prey of round goby, strongly reflect red light [122]. An enhanced red perception through an additional red opsin gene may thus be relevant for round goby predation success below 15 m. In turbid waters, red is the most common part of the light spectrum because long wavelengths experience least scattering [24]. Round gobies readily establish populations in turbid environments. The retention of two red opsin genes may thus possibly relate to the ability of the round goby to colonize turbid habitats. Our predictions based on the key amino acid substitution suggest that LWS1 is expected to be most sensitive at 560 nm (same as one of the mudskipper gobies) [28], while LWS2 is expected to be most sensitive at 550 nm [68]. Similar small differences in the sensitivity maximum can indeed result in functionally different spectral tuning palettes (e.g., during development or in different environmental conditions) [123], and we propose functional follow-up studies on the ability of round goby to deal with and perceive various red light conditions.

The presence of red fluorescence on top of the eye in round goby is the first unequivocal description of fluorescence in a freshwater fish and might be interpreted as being associated with the ability to discriminate different shades of red colors. However, the fluorescence in the specimens investigated was quite weak. Unless fluorescence expression is stronger under natural conditions or in the ancestral population from which the invading populations stem, a visual function of the weak fluorescence observed here seems unlikely (see warnings [124]). Fluorescence is, however, widespread and stronger among several marine gobies [69]. Although the fluorescent “eyebrows” of the round goby show a striking similarity to those of some marine gobies, their function will remain unclear until properly tested. Social functions are possible—for example, in sand gobies, dark eyes indicate female readiness to spawn [125]. Alternatively, they may simply provide camouflage for individuals buried in bright sand up to the eyes. Functional hypotheses for fluorescence, such as communication, camouflage, and improved prey detection have been extensively reviewed [126]. The genetic tools now available for the round goby may allow for experimental manipulation of fluorescence expression, once the actual fluorophores that produce the fluorescent signal have been identified.

### Response to the environment

With respect to ecological and physiological aspects that are related to the ability of species to deal with novel environments, some findings on CYP genes, on osmoregulation, and on innate immunity call for further attention. The mostly minimal complement of cytochrome P450 proteins present in the round goby is unexpected considering the occurrence of round goby in polluted areas [127, 128]. The CYP1–3 gene complement for xenobiotic metabolism is similar to other teleost genomes, and the ability of the round goby to survive in contaminated environments must therefore have other reasons. Round goby may cope with contaminations at the level of gene expression, either through higher basal expression values or by a particularly rapid response to exposure [49]. Alternatively, this species may have peculiarities in other, not yet analyzed areas of the defensome (e.g., transporters). Analyses of the tissue expression of CYP families 1, 2, and 3, and also the study of other defensome gene families, including the nuclear receptors regulating CYP gene expression, transporters, and conjugating enzyme families, may be useful in this respect.

Another potentially relevant finding is the ability of the round goby to not only produce, but also accumulate osmolytes. Species distribution constraints often arise from physiological limitations. The round goby is one of the most geographically wide-ranging invasive fish species in Europe and North America, and its predicted (although untested) ability to accumulate osmolytes may impact its range expansion in three ways. Firstly, 0–25 PSU (common for coastal waters, but lower than the ocean) is the species’ current limit for unperturbed osmoregulation [129]. However, the round goby’s repertoire of key genes in myo-inositol production and accumulation identified in this study might bestow the species with the potential to eventually tolerate higher salinities, for example through the evolution of altered gene regulation patterns, and colonize higher PSUs. Secondly, osmolytes improve water retention and thus desiccation tolerance. In this context, myo-inositol accumulation may have contributed to overland dispersal. Overland dispersal of fish eggs or larvae with boats or fishing gear involves air exposure, and indeed, round goby eggs withstand desiccation for up to 48 h [130]. Finally, osmolytes essentially act as anti-freeze agents and molecular chaperones and contribute to cryoprotection in diverse organisms from bacteria [131] to flies [132]. The surprising and unexpected ability of the round goby to colonize cold areas well below its temperature optimum of 22 °C, such as the Northern Baltic Sea, may be linked to osmolyte production. It remains to be tested whether osmolytes play a role in enabling the round goby to combat a number of environmental conditions and to colonize new areas.

Lastly, we observe copy number expansions/duplications in several round goby innate immunity gene families. Many of the duplications concern genes essential for inflammasome assembly, activation, and function. The fish inflammasome complex is somewhat poorly characterized, and maturation of IL-1 by inflammasome-activated Caspase 1 cleavage is a matter of debate because teleost IL-1 proteins lack the conserved caspase cleavage site present in mammalian IL-1b and IL-18 [133]. However, zebrafish (*Danio rerio*) Caspase 1 can utilize an alternative site to cleave and mature IL-1 [107, 134], and the presence of components such as ASC, caspases, and pro-IL1 and pro-IL18 further supports a role for inflammasomes in fish, particularly since zebrafish (*Danio rerio*) ASC oligomerize and form “specks” as seen in mammals [107]. The round goby with its strong inflammasome system may therefore present an attractive system to explore the molecular dynamics of inflammasome activation in fish.

In terms of survival in novel environments, the round goby’s expanded repertoire of pathogen recognition receptors may broaden the scope of its immune response and increase the range of detectable ligands and pathogens. Additionally, the expanded acute phase repertoire may contribute to a faster response. Inversely, the expansion may limit excessive cell damage during inflammation. In humans, the acute phase protein CRP contains inflammation as part of a negative feedback loop [135]. Thus, the round goby may re-enter homeostasis faster compared to other fish species with smaller CRP/APCS repertoires. The larger acute phase repertoire may also function to limit the cellular damage caused by the potentially large amount of inflammasome combinations the round goby can generate. In this context, we suggest systematic investigations into a potential relation between inflammasome expansions and invasiveness in *Gobiidae*, in combination with immune challenge experiments.

### Long-term adaptation

We identify a potentially interesting evolutionary history for the conserved PRC2 component EZH in fish and add to the previous observation that the conserved de novo DNA methylation machinery features a surprising diversity in fish. These results underscore the need for in-depth investigations into the role and relevance of epigenetic regulation and transgenerational inheritance in teleosts. Our findings support the emerging idea that epigenetic regulation in fish follows somewhat different rules than in mammals. For the histone-methylating complex PRC2, our results suggest interesting phylogenetic relationships of EZH proteins in fish. EZH proteins act in tissue-specific complexes comprised of core SUZ12, EED, and RBBP4, but also AEBP2, PCL proteins, and JARID2. These proteins enhance PRC2 efficiency, contribute to recruitment to target sites, or inhibit the complex [54, 109]. Small changes in amino acids can have strong effects on the complex, since the precise interactions among the components and with other gene regulators impact its function and localization [136–139]. For example, species-specific insertions [140] are thought to regulate PRC2 recruitment and/or exclusion from target genes [141]. We suggest that the future incorporation of more sequences of both EZH1 and EZH2 from a greater range of taxa and the inclusion of currently unannotated versions of the genes associated with both the teleost-specific whole-genome duplication and lineage-specific duplications [113] would aid understanding of the evolutionary history of the entire complex. We also expect that studying PRC2 in non-mammalian vertebrates may reveal ancestral or less abundant interactions, functions, or also complex associations of PRC2.

Similarly, our results warrant an in-depth exploration of DNA methylation in fish. Originally, DNA methylation evolved to distinguish own (methylated) DNA from foreign (non-methylated) DNA such as introduced by viruses. Therefore, cytosines in CG base contexts are by default methylated. In mammals, DNA methylation in CG dense regions (CG islands) is associated with gene repression. However, DNA methylation also features species- and taxon-specific differences, even among vertebrates, which are still greatly underappreciated. For example, non-methylated genome regions in fish are unexpectedly CG-poor [142], fish differ from mammals with respect to the distribution of methylated CpGs in the genome [143], algorithms developed on mammals fail to identify CpG islands in fish [144], genome-wide CpG island predictions in cold-blooded animals consist primarily of false positives [145], and fish CG methylation occurs mainly in coding regions, where it correlates positively with gene expression levels [146]. DNA methylation dynamics in the germline follows distinct and non-mammalian patterns in zebrafish [147, 148], mangrove fish [149], and medaka [150], and copy number variations in the de novo DNA methyltransferase DNMT3 in teleosts do not reflect teleost genome duplication events [116]. Together with distinct spatiotemporal expression patterns particularly during development [151–154], the peculiarities of the fish DNA methylation machinery clearly warrant an in-depth and species-aware exploration of the role of DNA methylation in fish.

### Gene expansions

A general theme across several of the analyzed gene families is gene expansions. Gene expansions are recurrent in fish genomes, both within [155, 156] and outside [157–159] the context of whole-genome duplications. For example, the copy number of immune genes in fish is quite plastic. Cod has disposed of some core adaptive immunity components [11], yellow croaker features an expanded TNF repertoire [160], and channel catfish (*Ictalurus punctatus*) retains a high number of recent duplications and SNPs in immune genes [159], while genes specifically retained after the fourth whole-genome duplication in salmonids are not immune genes [155]. Duplicated genes are known to experience rapid neofunctionalization rather than subfunctionalization [156], and have the potential to compensate against mutation even after divergence [161]. The process of gene duplication itself has long been considered to be one of the main sources of genetic variation and to have an adaptive potential [162], and in invertebrates, gene expansions have been explicitly linked to invasive potential [163, 164]. We observe a similar correlation, i.e., expansions are more prevalent in the analyzed gene families in the invasive round goby than in the closest genome-sequenced relative, the non-invasive goby species *B. pectinirostris*. Together, the *Benthophilinae* subgroup of *Gobiidae* is recently diversified crowd of fish with many members inexplicably moving into novel ecosystems [165], and Gobiidae in general share a remarkable colonization potential [17, 166]. Final conclusions about the contribution of duplications to round goby invasiveness cannot be drawn until additional related invasive and non-invasive goby species (e.g., sand goby and/or monkey goby) have been sequenced and comparatively analyzed. However, the round goby and its relatives are definitely strong candidates for a more systematic investigation of a potential link between gene expansions and invasiveness or colonization of novel environments in vertebrates in the future. Importantly, recent gene expansions can be difficult to resolve with short reads, and genomes based on long-read sequencing (as presented here) will be instrumental in this regard.

Among the receptor families analyzed, the NLRs, TLRs, and olfactory receptors, we identify a couple of particularly beautiful case studies for recent expansions and repeated radiations. Our identification of two previously undescribed NLR-C gene families [102], here termed group 5 and group 6, in *Procanthopterygii*, and their absence in *Otocephala* that are separated by 230 million years, indicates substantial diversification of NLRs between major fish lineages and warrants an in-depth investigation of evolutionary processes generating NLR diversity in fish.

In addition, the observed cases of gene expansions in receptors warrant investigations into the evolution of ligand binding repertoires. For example, olfactory receptor 7tm1 subfamily members are expanded in *Gobiidae*, and we hypothesize that they may be involved in the detection of distinctive types of odors relevant for *Gobiidae* ecology and/or reproduction [31–33]. Which types of odorants are detected by parallel expanded ORs, and whether these expansions serve to detect similar or different types of odorant molecules in different species, remain to be studied. Similarly, the massively expanded TLR22 and TLR23 families warrant an exploration of their ligand binding properties. TLR22 is upregulated in channel catfish (*Ictalurus punctatus*) upon bacterial infection [97] and both receptors have been suggested to recognize nucleic acid ligands [98], but some also react to protein or lipid pathogen-associated patterns [167–169]. Their role in fish is currently unclear. Analyses of tissue-specific expression, as has been attempted for some NLR genes [104, 106, 170, 171], may be a useful approach to elucidate the functional significance of large receptor repertoires.

In summary, this work provides a solid basis for future research on the genomic, genetic, and epigenetic basis of species persistence in the face of change, and of the ability to colonize a novel environment. Clearly, many more gene families or pathways are expected to contribute to the ability of round goby to invade ecosystems and outcompete related or ecologically similar species. For example, the presented analyses barely scratch the surface of epigenetic regulation, innate immunity, and transporters (e.g., of toxins). We did not investigate endocrine pathways (which govern growth and reproductive success) nor antimicrobial peptides (which contribute to innate immune defense), areas which may yield fruitful information of the success of this invader. We welcome future research using this novel genomic resource and encourage experts on those pathways to contribute their knowledge.

## Methods

A relevant note upfront the methods section is that this manuscript is the product of a long-standing collaboration of experts in their respective fields. The gene families analyzed differ widely with regard to sequence conservation, the number and similarity of genes within and between species, the scope of questions in the field, etc. Compare, for example, the de novo identification of hundreds of virtually identical NLR receptors with the manual annotation of a handful of extremely conserved DNA methyltransferases, or the phylogenetic analysis of the conserved vertebrate CYP gene family with a fish-centered comparison of osmotic balance regulators which are very different in scope and necessary phylogenetic resolution. Accordingly, each author applied methods that were suited for the respective situation. As a common theme, genes were identified by blast, sequences were extracted and aligned with other fish and/or other vertebrates, trees were constructed with either Bayesian or maximum likelihood methods, and findings were always verified against the mudskipper genomes.

### Genomic DNA library preparation and PacBio sequencing

Genomic DNA was extracted from the liver of one male individual of round goby caught in Basel, Switzerland (47° 35′ 18″ N, 7° 35′ 26″ E). At the Genome Center Dresden, Germany, 300 mg of frozen liver tissue was ground by mortar and pestle in liquid nitrogen and lysed in Qiagen G2 lysis buffer with Proteinase K. RNA was digested by RNase A treatment. Proteins and fat were removed with two cycles of phenol-chloroform extraction and two cycles of chloroform extraction. Then, DNA was precipitated in 100% ice-cold ethanol, spooled onto a glass hook, eluted in 1× TE buffer, and stored at 4 °C. A total of 10 μg of DNA was cleaned using AMPure beads. From this DNA, five long insert libraries were prepared for PacBio sequencing according to the manufacturer’s protocols. Genomic DNA was sheared to 30–40 kb using the Megaruptor device. The PacBio libraries were size selected for fragments larger than 15–17.5 kb using the BluePippin device. PacBio SMRT sequencing was performed with the P6/C4 chemistry using 240 min sequencing runs. Average read length was 11–12 kb. In total, 86 SMRT cells were sequenced on the PacBio RSII instrument resulting in 46 gigabases (Gb; an estimated 46× coverage for a putative ~ 1 Gb genome) polymerase reads.

### Assembly of the round goby genome

The round goby genome was assembled at the Heidelberg Institute for Theoretical Studies HITS gGmbH. Raw PacBio reads were assembled using the Marvel assembler [172, 173] with default parameters unless mentioned otherwise. Marvel consisted of three major steps, namely the setup phase, patch phase, and the assembly phase. In the setup phase, reads were filtered by choosing only the best read of each Zero-Mode Waveguide as defined by the H5dextract tool [172] and requiring subsequently a minimum read length of 4k. The resulting 3.2 million reads were stored in an internal Marvel database. The patch phase detected and fixed read artifacts including missed adapters, polymerase strand jumps, chimeric reads, and long low-quality segments that are the primary impediments to long contiguous assemblies [172]. To better resolve those artifacts, only low complexity regions were masked with the Dbdust command and no further repeat masking was done. The resulting patched reads longer than 3k (41× coverage) were then used for the final assembly phase. The assembly phase stitched short alignment artifacts from bad sequencing segments within overlapping read pairs. This step was followed by repeat annotation and the generation of the overlap graph, which was subsequently toured in order to generate the final contigs. By using an alignment-based approach, the final contigs were separated into a primary set and an alternative set containing bubbles and spurs in an overlap graph. To correct base errors, the correction module of Marvel was used in an initial step, which made use of the final overlap graph and corrected only the reads that were used to build the contigs. After tracking the raw reads to contigs, PacBio’s Quiver algorithm [174] was applied twice to further polish contigs as previously described [172].

### Automated annotation of the round goby genome

The round goby genome assembly was annotated using Maker v2.31.8 [175, 176]. Two iterations were run with assembled transcripts from round goby embryonic tissue [50] and data from 11 other actinopterygian species available in the ENSEMBL database [177] (downloaded the 15th February 2016, see Table 5) as well as the SwissProt protein set from the uniprot database as evidence [178] (downloaded March 2, 2016). In addition, an initial set of reference sequences obtained from a closely related species, the sand goby (*Pomatoschistus minutus*), sequenced by the IMAGO marine genomes project of the CeMEB consortium at University of Gothenburg, Sweden, was included. The second maker iteration was run after first training the gene modeler SNAP version 2006-07-28 [179] based on the results from the first run. Augustus v3.2.2 [180] was run with initial parameter settings from zebrafish (*Danio rerio*). Repeat regions in the genome were masked using RepeatMasker known elements [181] and repeat libraries from Repbase [182] as well as de novo identified repeats from the round goby genome assembly obtained from a RepeatModeler analysis [181].

**Table 5:** Annotation reference data

Reference species	Number of protein sequences	Assembly version from ENSEMBL (downloaded 15th Feb 2016)
*Astyanax mexicanus*	23,698	AstMex102
*Danio rerio*	44,487	GRCz10
*Gadus morhua*	22,100	gadMor1
*Gasterosteus aculeatus*	27,576	BROADS1
*Lepisosteus oculatus*	22,483	LepOcu1
*Oreochromis niloticus*	26,763	Orenil1.0
*Oryzias latipes*	24,674	MEDAKA1
*Poecilia formosa*	30,898	PoeFor_5.1.2
*Takifugu rubripes*	47,841	FUGU4
*Tetraodon nigroviridis*	23,118	TETRAODON8
*Xiphophorus maculatus*	20,454	Xipmac4.4.2

Summary of reference data from Ensembl used for the annotation

In order to ensure the completeness and quality of the current assembly and the associated gene models, the assembly and the predicted protein sequences were run against reference sets at two different taxonomical levels (303 eukaryotic and 4584 actinopterygian single-copy orthologues) using the BUSCO pipeline v2.0 [183, 184].

The maker annotation results were used to generate a database for JBrowse/Webapollo using the script “maker2jbrowse” included with JBrowse [185, 186]. Predicted protein and transcript sequences were used to query the uniprot database, using blastp and blastn respectively, and the best hit descriptions were transferred to the fasta headers with scripts bundled with Maker as described in [176].

### Sex-determining regions

To investigate whether the round goby genome features large sex-determining regions, own available RAD sequencing data were analyzed. Restriction site-associated DNA (RAD) [187] libraries were prepared following a protocol used by Rösti et al. [188, 189], which is largely based on Hohenlohe et al. [190]. In short, the Sbf1 enzyme was used on DNA extracted from 57 females, 56 males, and 5 juveniles caught in Basel, Switzerland, and 39–40 individuals were pooled per library for SR 100 bp sequencing with Illumina (raw reads deposited at SRA [REF]. In total, 45 females and 47 males retained sufficient numbers of reads (> 150,000) per sample after cleaning and demultiplexing, were processed with the Stacks pipeline using the genome-independent approach [191], and were analyzed for sex-specific loci present exclusively in males or females. Considering a genome size of ~ 1 GB, the presence of 23 chromosomes [192], and a calling success of 21,877 loci in 95 or 96 individuals (49,220 loci in at least 40 individuals), an average density of one RAD locus every 45,710 (20316) bp and an average number of 951 (2140) markers is expected for an average sized chromosome. The presence of a sex chromosome should thus be indicated by hundreds of sex-specific RAD loci, while a contiguous sex-determining region larger than 45,000 bp would be indicated by one or more sex-specific RAD loci. Read numbers per locus for each sample were extracted from the *.matches.tsv file output from Stacks and analyzed for sex-specific loci with standard R table manipulation.

### Vision

Opsin genes were extracted from the genome assembly using the Geneious software [193] by mapping the genomic scaffolds (settings: medium sensitivity, 70% identity threshold) against individual opsin exons of nile tilapia (*Oreochromis niloticus*; GenBank Acc. no.: MKQE00000000.1). This led to capturing of all scaffolds containing any visual opsin. The genes were then annotated by mapping back of the single exons of nile tilapia (*Oreochromis niloticus*) against each scaffold separately (settings: high sensitivity; 50% identity threshold) combined with the Live Annotate & Predict function as implemented in Geneious [193], based on the nile tilapia (*Oreochromis niloticus*) and various mudskipper species [28] opsin gene annotation. All regions upstream and downstream from every opsin gene, as well as the intergenic regions, were separately tested for presence of any further opsin gene or its fragment (pseudogene). The annotated genes were checked for the reading frame and the putative protein product was predicted.

Phylogenetic analysis on the visual opsin genes (i.e., SWS1, SWS2, RH2, RH1, and LWS opsins) were then performed across vertebrates, with focus on selected model species of teleost fishes. Special focus was laid on the LWS genes from the fish species or lineages known to possess multiple LWS copies, such as livebearers and pupfishes (Cyprinodontiformes) [194], zebrafish (*Danio rerio*) [195], salmon (*Salmo salar*) [196], common carp (*Cyprinus carpio*) [196], cavefish (*Astyanax mexicanus*) [197], Northern pike (*Esox lucius*) [196], labyrinth fishes (*Anabas testudineus*) [23], Asian arowana (*Scleropages formosus*) [196], and other gobies, such as mudskippers [28] and reef gobies [23]. The opsin gene sequences from round goby and other fish species, including outgroup of non-visual opsins (pinopsin, parietopsin, vertebrate-ancestral opsins and opn3 opsin; see Additional file 21 for alignment input fasta sequences), were aligned using the MAFFT [198] plugin (v1.3.5) under the L-ins-i algorithm as implemented in Geneious. Exon 5 (exon 6 in case of LWS) and part of exon 1 (or entire exon 1 in case of LWS), which provided ambiguous alignment due to their higher variability, were discarded. Model parameters were estimated by jModeltest 2.1.6 [199, 200], and subsequently Bayesian inference was used to calculate single-gene phylogeny using the MrBayes 3.2.6 [201] software as implemented on the CIPRES Science gateway [202].

### Fluorescence

Fish were illuminated with a Hartenberger mini compact LCD dive torch, equipped with custom-ordered green LEDs. A green laser clean-up filter (HC Laser Clean-up MaxLine 532/2, transmission 530–534 nm) was attached to the front and limited the emitted light to a 5-nm band around 532 nm. Pictures were taken with a Nikon D700 with a 105-mm Nikkor macro-lens through a single notch filter (ZET532NF nm, diameter 60 mm, AHF AG). This filter blocks out the 525–545-nm range and thereby completely suppresses the green excitation light. White balance was post-processed using Nikon ViewNX.

### Olfaction

Olfactory receptor (OR) peptide sequences to be used as query were extracted from a publicly available nile tilapia (*Oreochromis niloticus*) protein dataset [203]. Those queries were blasted (tblastn) against the genomes of the round goby (*Neogobius melanostomus*), the blue-spotted mudskipper (*Boleophthalmus pectinirostris*) [28], the giant mudskipper (*Periophthalmodon magnuspinatus*) [28], and the threespine stickleback (*Gasterosteus aculeatus*) [204], using an *e*-value threshold of 10e^− 50^. Only the hit with the highest bit-score for each genomic position with more than one alignment was employed in subsequent steps. Mapped hits belonging to contiguous positions of the protein (maximum overlap of 15 amino acids) and with a genomic distance smaller than 10 kb were joined as exons of the same CDS-gene model. Obtained sequences were translated to proteins using TransDecoder [205], filtering all models that produce peptides smaller than 250 amino acids. While many ORs are usually around 300 amino acids long in total, 250 is close to the average size of their main transmembrane domain, which is centrally located in the protein and more suitable to interspecific alignment compared to N-terminal and C-terminal ends. Accordingly, this method might introduce a reduced proportion of recent pseudogenes that could lead to a small overestimation of OR genes with coding capacity, although all species should be affected equivalently.

Next, an hmmscan [206] was produced against Pfam database to identify the domain with the highest score for each obtained protein sequence. False positive detection was filtered against using blast against confident OR and non-OR protein datasets. For phylogenetic analysis, sequences (see Additional file 22 for fasta sequences) were aligned with MAFFT [207] and a Maximum Likelihood methodology was employed to build the tree using W-IQ-TREE software [208] with standard parameters and Ultrafast bootstrap [209]. Four adrenergic receptor sequences from nile tilapia (*Oreochromis niloticus*) were used as an outgroup. Monophyletic groups formed by five or more genes of the same species were considered as lineage-specific gene expansions. Because of the phylogenetic proximity of the two mudskippers and the differences in their genome assembly statistics, only blue-spotted mudskipper (*Boleophthalmus pectinirostris*) was considered and sequences from the mudskipper species *Periophthalmus magnuspinnatus* were allowed to be included in their lineage-specific expansion groups.

### Detoxification

The Basic Local Alignment Search Tool (BLAST, v. 2.2.31) [210] was used to identify local alignments between the round goby genome and a query including all annotated CYPs in humans (*Homo sapiens*) and zebrafish (*Danio rerio*; vertebrate) and the most dissimilar invertebrate CYPs from arthropod fruitfly (*Drosophila melanogaster*), nematode *Caenorhabditis elegans*, and the annelid worm *Capitella teleta* (see Additional file 23 for fasta query sequences). Only BLAST high scoring pairs with expect values of 1.0 × 10–10 or smaller were considered significant.

The JBrowse genome viewer (v1.12.1) [211] was used to manually annotate the significant regions of each genome from the BLAST search, identifying start (ATG) and stop (TGA/TAA/TAG) codons, exon number, and splice site signals (GT/AG) at intron-exon boundaries. The lengths of the potential CYPs were identified and considered full length at ~ 500 amino acid residues long. Potential genes were matched to the well-curated cytochrome P450 HMM in the Pfam protein family database [212] to confirm identity. The ScanProsite tool [213] was used to verify the presence of four largely conserved CYP motifs: the I-helix, K-helix, meander coil, and heme loop. Each gene was classified as “complete” (proper length with start and stop codon, all motifs present, and match to the HMM) or “partial” (presence of at least the entire ~ 120 amino acid region that contains all motifs but clearly less than full length). Any potential CYP that was missing at least one of the motifs was considered a gene “fragment” (see Additional file 5: Table S2 for names, sequences, and genomic locations the identified complete, partial, and fragment genes).

All “complete” and “partial” round goby CYPs (Additional file 5: Table S2) were included in further analyses. Clustal Omega (v1.2.4) [214] was used to generate a multiple sequence alignment of the round goby sequences and a variety of well-known vertebrate CYPs from human (*Homo sapiens*), zebrafish (*Danio rerio*), mouse (*Mus musculus*), frog (*Xenopus laevis*), chicken (*Gallus gallus*), and rat (*Rattus norvegicus*; 125 sequences in total; see Additional file 24 for fasta sequences). Mesquite (v3.10) [215] was utilized to trim the alignment, especially at the termini of the protein sequences where significant variation is typically observed, leaving only the portion of the alignment representative of the homology of the sequences. The final “masked” alignment (provided in Additional file 25) was used as input for the Randomized Axelerated Maximum Likelihood program (RAxML v8.2.10) [216]. A total of 100 bootstrap trees were generated with the rapid generation algorithm (−x) and a gamma distribution. The JTT substitution matrix with empirical frequencies was implemented in tree generation. The final maximum likelihood phylogenetic tree was visualized with FigTree (v1.4.3) [217] and rooted with the CYP51 family of enzymes.

### Osmoregulation

Protein sequences for aquaporins, tight junction proteins, ion transporters, and enzymes in osmolyte production pathways were retrieved from the round goby genome by BLASTing well-characterized proteins from zebrafish (*Danio rerio*), downloaded from Uniprot [178] (March 2018), against the round goby gene models/proteins. Only round goby gene models/proteins for which the predicted protein covered at least 70%, with a sequence identity of at least 40% and with *e*-value < 10^− 20^ of the corresponding protein in zebrafish (*Danio rerio*) were used for the phylogenetic analyses. Well-established paralogues belonging to different subclasses of the respective protein family, based on either literature search or from initial phylogenetic analysis of that particular protein family, were used as additional query sequences to minimize the risk of missing relevant round goby sequences. Osmoregulatory genes from human (*Homo sapiens*) and zebrafish (*Danio rerio*) were used for overall classification of clades in the respective protein family. Some manual curation was applied to the retrieved round goby sequences before analysis to correct for misannotations during automated gene prediction: (i) For NHE ion transporters, a 780-aa long non-homologous N-terminus from one of the *Neogobius* sequences was removed before the phylogenetic analysis. (ii) Some of the claudin genes were subjected to manual curation of Maker-predicted proteins. The claudin genes in fish consist of several tandem arrays, which in some cases results in merging of 2–4 claudin genes by the Maker software. Claudins have a typical transmembrane (TM) pattern with four distinct TM domains. All manually curated claudin genes from round goby were examined to have the expected four TM domains by TMHMM searches. Round goby protein sequences after manual curation are available in Additional file 26.

No myo-inositol phosphate synthase (MIPS) and sodium/inositol co-transporter (SMIT) proteins from zebrafish (*Danio rerio*) were found in Uniprot [178]. To confirm that there are truly no MIPS and SMIT genes in zebrafish (*Danio rerio*), the zebrafish genome GRCz11 at NCBI was also searched for homologies using blastp and tblastn using as query the MIPS and SMIT protein sequences from nile tilapia (*Oreochromis niloticus*) as query, and no hits were found. Thus, in the case of MIPS and SMIT, nile tilapia (*Oreochromis niloticus*) sequences were used for searching for round goby (*Neogobius melanostomus*) homologs. For the phylogenetic analyses, protein sequences from zebrafish (*Danio rerio*; except for MIPS and SMIT, see above), threespine stickleback (*Gasterosteus aculeatus*), nile tilapia (*Oreochromis niloticus*), blue-spotted mudskipper (*Boleophthalmus pectinirostris*), and human (*Homo sapiens*; exception for human NKA-beta) were used in comparison to round goby and were obtained from Uniprot [178] (zebrafish, threespine stickleback, nile tilapia, human) or RefSeq [218] (mudskipper; see Additional file 26 for fasta sequences). Phylogenetic analyses of osmoregulatory proteins in round goby were performed using maximum likelihood with the PhyML v3.0 [219] online tool with 100 bootstraps and using Gblocks to eliminate poorly aligned positions and highly divergent regions.

### Immune system

To perform an overall characterization of key genes related to the immune system, protein queries representing core components of innate and acquired immunity from several fish species as well as mammalian reference sequences were downloaded from UniProt [178] and Ensembl [177]. The protein queries were aligned prior to usage to ensure sequence homology. Previously extracted protein sequences from the Toll-like receptor family [98], and MHCI sequences [91] were also used as query. All queries are listed in Additional file 11: Table S4. To enable comparative analyses between sequenced Gobiiformes, the genomes of the mudskipper species *Periophthalmodon schlosseri* (GCA_000787095.1), *Periophthalmus magnuspinatus* (GCA_000787105.1), *Scartelaos histophorus* (GCA_000787155.1), and *Boleophthalmus pectinirostris* (GCA_000788275.1) were additionally downloaded from NCBI.

All protein queries were used in a tblastn (blast+ v. 2.6.0) towards the round goby genome assembly using default parameters and a *e*-value cutoff of 1e−10 [220]. Some queries (*caspase-1*, *TLRs*, *IL1*, and *IL8*) were also used in an identical tblastn towards the other Gobiiformes genomes. Genomic hit regions were extracted using BEDtools (v. 2.17.0) [221] extending both up- and downstream as needed to obtain full-length gene sequences. The extracted genomic regions were imported into MEGA7 [222, 223]; the reading frame was adjusted for each exon and aligned as proteins to the corresponding translated coding sequence of queries using MUSCLE [222, 223] with default parameters. Intronic sequences were removed leaving an in-frame coding sequence. All alignments were subjected to manual evaluation before subsequent analysis.

To generate phylogenetic trees, protein alignments were made and model tested using the ProtTest3 server [224] specifying BIC and no tree optimization (server has been disabled but ProtTest is available for download from GitHub). All alignments reported the JTT model as best hit. Maximum likelihood trees were produced by using RAxML-PTHREADS (v 8.0.26) [225], PROTCATJTT, rapid bootstrap, and 500 bootstrap replicates. The final trees were imported into FigTree [217] (http://tree.bio.ed.ac.uk/software/figtree/), and subsequently Adobe Illustrator for presentation purposes.

In order to identify members of the large multigenic family of fish-specific NACHT and Leucine-Rich Repeats containing genes (NLRs; the fish-specific subset is also known as NLR-C) [101], an alignment of 368 zebrafish (*Danio rerio*) NLR-C proteins [102] was used as query. A combination of tblastn, HMMER3 searches [226], and alignments with MAFFT v7.310 [227] was used to generate first an initial list of “candidate regions” potentially containing an NLR (see Additional file 27 for fasta sequences of candidate regions) and then an annotation of the characteristic domains in round goby NLR-C family members (see Additional file 28 for detailed methods and Additional file 17: Table S8 for genomic location of annotated domains), consisting of 25 PYRIN, 1 N-terminal CARD, 12 C-terminal CARD, 343 FISNA-NACHT, and 178 B30.2 domains. Custom HMM models for major NLR exons (FISNA-NACHT, and PRY-SPRY/B30.2) were generated and utilized during this process (see Additional file 29 for hmm models). The majority of identified FISNA-NACHT exons contained frameshifts or a large insertion, indicating either pseudogenization, acquisition of new introns, problems with the assembly, or a combination of the three [228]. For subsequent phylogenetic analyses, only the 61 clearly intact NLRs were used. These were aligned with NLRs from human (*Homo sapiens*), zebrafish (*Danio rerio*), and the blue-spotted mudskipper (*Boleophthalmus pectinirostris*) using MAFFT [207] (see Additional file 28 for detailed methods and Additional file 30 for alignments); maximum Likelihood trees were produced with RAxML-PTHREADS, PROTCATJTT, rapid bootstrap, and 500 bootstrap replicates [225]. The final trees were imported into FigTree [217] (http://tree.bio.ed.ac.uk/software/figtree/), and subsequently Adobe Illustrator. The alignments were inspected manually for presence of the conserved Walker A motifs and sequence logos for these were generated with WebLogo [229]. Finally, a survey of the PYD domains, Peptidase_C14 domains (Caspases), and CARD domains was performed. All cases of a PYD domain followed by an adjacent CARD in the round goby (putative apoptosis-associated speck-like protein containing a CARD (ASC), also known as PYD-CARD or PYCARD) were identified from the HMMER3 dataset. The open reading frames containing these were translated, concatenated, and aligned with similarly structured proteins from human (*Homo sapiens*), mouse (*Mus musculus*), lizard (*Anolis carolinensis*), frog (*Xenopus laevis*), and all the fish in Ensembl, and with PYD-CARDs identified from the other available goby assemblies (see Additional file 31 for aligned sequences). A phylogenetic tree was generated as described above. The annotation for NLR-C genes consists of predicted positions for all of the major conserved NLR-associated domains (PYD, CARD, FISNA-NACHT helices, LRRs, B30.2; see Additional file 17: Table S8 for genomic location of annotated domains). To post hoc add miiuy croaker (*Miichthys miiuy*), the FISNA-NACHT exon was extracted from the respective genomes with the custom HMM model and added to the existing NLR alignment with *mafft –add –keeplength*. The resulting file was manually trimmed to start with the FISNA domain and end at the conserved last amino acid of the exon. For the channel catfish (*Ictalurus punctatus*), the available mRNA sequences for NLR genes were translated with EMBOSS transeq [230] and aligned as above. The tree itself was generated with RAxML-PTHREADS [225], PROTCATJTT, rapid bootstrap, and 100 bootstrap replicates.

### Epigenetic regulators

The focus of this analysis was on two conserved eukaryotic gene expression regulators: the Polycomb Repressive Complex 2 (PRC2), which deposits repressive histone methylation marks, and the DNA methyltransferases, which methylate cytosine in CpG contexts. The presence of both marks is associated with a downregulation of gene expression, mostly by extrapolation from mammals and plants. The protein sequences of zebrafish (*Danio rerio*) orthologues of PRC2 components RBBP2, EED, EZH1-2, and SUZ12 [54] and of DNA methylases DNMT1 and DNMT3 [231] were blasted against the round goby genome using default parameters at a Blast server intermittently hosted by the University of Gothenburg. The protein sequence of predicted proteins at the hit site was extracted manually in the round goby genome browser and aligned with mouse (*Mus musculus*), human (*Homo sapiens*), and zebrafish (*Danio rerio*) protein sequences. When the first and/or last exon sequences as predicted in the round goby genome differed significantly from the mouse (*Mus musculus*), human (*Homo sapiens*), and zebrafish (*Danio rerio*) sequences, confirmation by 3′ and 5′ RACE was attempted on RNA extracted from whole juvenile animals (see Additional file 32 for a detailed RACE protocol). A putative CDS was combined from automated annotation and RACE results, and aligned to sequences extracted from a variety of fish taxa, shark, chicken, frog, lizard, and human (see Additional file 33 for alignment files) with codon aware alignment MACSE [232]. Given the high conservation of these proteins among eukaryotes, and the absence of major unexpected differences between round goby and other vertebrates, additional Gobiidae were not included in the analyses. The model and partitioning scheme used for each phylogenetic analysis was estimated using PartitionFinder2 [233] using PhyML [219] with corrected AIC scores (AICc) used for model selection. Phylogenetic analyses were performed using MrBayes 3.2.6 [201, 234] with three independent runs for each gene. Analyses were run for 2,000,000 generations or until the standard deviation of split frequencies was below 0.01 up to a maximum of 20,000,000 generations. In order to aid convergence in the EZH analyses, the temperature parameter was set to 0.05.

### Transposable elements

A number of different applications were used for the repeat annotation of the genome. They are described in the repeat annotation report in Additional file 34. In summary, in addition to the identification of repeats with RepeatModeler [235] as described above, TRF [236] was used to predict tandem repeats. RepeatMasker [181], a homology-based approach, was used to produce a genome-wide overview of interspersed repeats. LTR Finder [237] and LTRharvest [238] in combination with LTRdigest [239], both de novo approaches, were used to predict LTRs.

## Additional files

Additional file 1: **Table S1.** Overview and results for repeat annotation.

Additional file 2: **Figure S1.** Phylogenetic tree of opsins, with the branches that are depicted collapsed in Fig. 2 expanded.

Additional file 3: **Figure S2.** Phylogenetic tree of opsins constructed with individual exons.

Additional file 4: **Figure S3.** Hylogenetic tree of olfactory receptors, with the brances that are depicted collapsed in Fig. 3 expanded.

Additional file 5: **Table S2.** Annotation of CYP genes identified in round goby.

Additional file 6: **Figure S4.** Phylogenetic tree of claudins.

Additional file 7: **Figure S5.** Phylogenetic tree of occludins.

Additional file 8: **Figure S6.** Phylogenetic trees of various sodium transporters.

Additional file 9: **Figure S7.** Phylogenetic trees of genes involved in myo-inositol production and accumulation.

Additional file 10: **Table S3.** Overview table of immune genes analysed.

Additional file 11: **Table S4.** Immune gene sequences used as query.

Additional file 12: **Table S5.** Annotation of MHCI genes identified in round goby.

Additional file 13: **Table S6.** Annotation of MHCII genes identified in round goby.

Additional file 14: **Figure S8.** Phylogenetic tree of TAP genes.

Additional file 15: **Table S7.** Annotation of other immune genes identified in round goby.

Additional file 16: **Figure S9.** Schematic of the immunoglobulin locus.

Additional file 17: **Table S8.** Annotation of NLR genes identified in round goby.

Additional file 18: **Figure S10.** Phylogenetic tree of Gobiidae TLRs.

Additional file 19: **Figure S11.** Phylogenetic tree of CRP / APCS.

Additional file 20: **Figure S12.** Phylogenetic trees of SUZ12, EED, and RBBP4.

Additional file 21. Opsin sequences used for tree building.

Additional file 22. Olfactory receptor sequences used for tree building.

Additional file 23. CYP sequences used as query.

Additional file 24. CYP sequences used for tree building.

Additional file 25. Alignment of CYP sequences.

Additional file 26. Osmoregulatory protein sequences used for tree building.

Additional file 27. NLR candidate regions.

Additional file 28. Detailed methods for NLR annotation.

Additional file 29. Hmm models used to identify NLRs.

Additional file 30. NLR sequences used for vertebrate tree building.

Additional file 31. NLR sequences used for Gobiidae tree building.

Additional file 32. Detailed methods for 3’ and 5’ RACE of epigenetic regulators.

Additional file 33. Alignments of dnmt1, dnmt3, eed, ezh, rbbp4, and suz12.

Additional file 34. Detailed methods for repeat annotation.

## Data Availability

The genome sequence has been deposited in the NCBI nucleotide database under the GenBank accession VHKM00000000 [60]. Annotation tracks have been deposited in the Zenodo database (zenodo.org) as “Supplemental_Material_S1_Round goby_Genome_Annotation.gz” under the DOI 10.5281/zenodo.3561919 [240]. The raw reads obtained from RAD sequencing have been deposited at the NCBI SRA database under NCBI BioProject PRJNA547536 [63]. Other raw read resources indicated in Table 1 (RNA sequencing of liver and embryos, brain DNA methylation, brain and liver ATAC sequencing) are also deposited at the NCBI SRA database [61–64]. All other dataset(s) supporting the conclusions of this article are included within the article and its additional files.

## References

[1] Prentis PJ, Wilson JR, Dormontt EE, Richardson D, Lowe AJ (2008). Adaptive evolution in invasive species. Trends Plant Sci.

[2] Tsutsui ND, Suarez AV, Holway DA, Case TJ (2000). Reduced genetic variation and the success of an invasive species. Proc Natl Acad Sci.

[3] Lee CE (2002). Evolutionary genetics of invasive species. Trends Ecol Evolution.

[4] Bock DG, Caseys C, Cousens RD, Hahn MA, Heredia SM, Hübner S (2015). What we still don’t know about invasion genetics. Mol Ecol.

[5] Jude DJ, Reider RH, Smith GR (1992). Establishment of Gobiidae in the Great Lakes Basin. Can J Fish Aquat Sci.

[6] Michalek M, Puntila R, Strake S, Werner M (2012). HELCOM Baltic Sea Environment Fact Sheet 2012: Helcom.

[7] Roche KF, Janač M, Jurajda P (2013). A review of Gobiid expansion along the Danube-Rhine corridor – geopolitical change as a driver for invasion. Knowl Manag Aquat Ecosyst.

[8] Hirsch Philipp E., N’Guyen Anouk, Adrian-Kalchhauser Irene, Burkhardt-Holm Patricia (2015). What do we really know about the impacts of one of the 100 worst invaders in Europe? A reality check. Ambio.

[9] Aparicio S, Chapman J, Stupka E, Putnam N, Chia J, Dehal P (2002). Whole-genome shotgun assembly and analysis of the genome of fugu rubripes. Science.

[10] Amemiya CT, Alföldi J, Lee AP, Fan S, Philippe H, MacCallum I (2013). The African coelacanth genome provides insights into tetrapod evolution. Nature.

[11] Star B, Nederbragt AJ, Jentoft S, Grimholt U, Malmstrom M, Gregers TF (2011). The genome sequence of Atlantic cod reveals a unique immune system. Nature.

[12] Li J, Hou G, Kong X, Li C, Zeng J, Li H (2015). The fate of recent duplicated genes following a fourth-round whole genome duplication in a tetraploid fish, common carp (*Cyprinus carpio*). Scientific Reports.

[13] van Kessel N, Dorenbosch M, Kranenbarg J, van der Velde G, Leuven R (2016). Invasive Ponto-Caspian gobies rapidly reduce the abundance of protected native bullhead. Aquat Invasions.

[14] Burkett EM, Jude DJ (2015). Long-term impacts of invasive round goby Neogobius melanostomus on fish community diversity and diets in the St. Clair River, Michigan. J Great Lakes Res.

[15] Števove B, Kováč V (2013). Do invasive bighead goby Neogobius kessleri and round goby N. melanostomus (Teleostei, Gobiidae) compete for food? Knowledge and Management of Aquatic. Ecosystems.

[16] Dufour BA, Hogan TM, Heath DD (2007). Ten polymorphic microsatellite markers in the invasive round goby (Neogobius melanostomus) and cross-species amplification. Mol Ecol Notes.

[17] Adrian-Kalchhauser I, Svensson O, Kutschera VE, Alm Rosenblad M, Pippel M, Winkler S (2017). The mitochondrial genome sequences of the round goby and the sand goby reveal patterns of recent evolution in gobiid fish. BMC Genomics.

[18] Feldheim KA, Willink P, Brown JE, Murphy DJ, Neilson ME, Stepien CA (2009). Microsatellite loci for Ponto-Caspian gobies: markers for assessing exotic invasions. Mol Ecol Resour.

[19] Neilson ME, Stepien CA (2009). Escape from the Ponto-Caspian: evolution and biogeography of an endemic goby species flock (Benthophilinae: Gobiidae: Teleostei). Mol Phylogenet Evol.

[20] Bowley LA, Alam F, Marentette JR, Balshine S, Wilson JY (2010). Characterization of vitellogenin gene expression in round goby (Neogobius melanostomus) using a quantitative polymerase chain reaction assay. Environ Toxicol Chem.

[21] Thacker CE, Roje DM (2011). Phylogeny of Gobiidae and identification of gobiid lineages. Syst Biodivers.

[22] Thacker CE, Thompson AR, Roje DM (2011). Phylogeny and evolution of Indo-Pacific shrimp-associated gobies (Gobiiformes: Gobiidae). Mol Phylogenet Evol.

[23] Musilova Z, Cortesi F, Matschiner M, Davies WIL, Patel JS, Stieb SM (2019). Vision using multiple distinct rod opsins in deep-sea fishes. Science.

[24] Seehausen O, van Alphen JJM, Witte F (1997). Cichlid fish diversity threatened by eutrophication that curbs sexual selection. Science.

[25] Seehausen O, Terai Y, Magalhaes IS, Carleton KL, Mrosso HDJ, Miyagi R (2008). Speciation through sensory drive in cichlid fish. Nature.

[26] Barth FG, Schmid A, Douglas RH (2001). The ecology of teleost fish visual pigments: a good example of sensory adaptation to the environment?: Ecology of Sensing.

[27] Hornsby MAW, Sabbah S, Robertson RM, Hawryshyn CW (2013). Modulation of environmental light alters reception and production of visual signals in Nile tilapia. J Exp Biol.

[28] You X, Bian C, Zan Q, Xu X, Liu X, Chen J, et al. Mudskipper genomes provide insights into the terrestrial adaptation of amphibious fishes. Nat Commun. 2014;5:5594. 10.1038/ncomms6594 PMC4268706 25463417

[29] Busserolles F de, Cortesi F, Helvik JV, Davies WIL, Templin RM, Sullivan RKP et al. Pushing the limits of photoreception in twilight conditions: The rod-like cone retina of the deep-sea pearlsides. Sci Adv. 2017;3(11):eaa04709. 10.1126/sciadv.aao4709 PMC5677336 29134201

[30] Kenaley CP, Devaney SC, Fjeran TT (2014). The complex evolutionary history of seeing red: molecular phylogeny and the evolution of an adaptive visual system in deep-sea dragonfishes (Stomiiformes: Stomiidae). Evolution.

[31] Corkum LD, Arbuckle WJ, Belanger AJ, Gammon DB, Li W, Scott AP (2006). Evidence of a male sex pheromone in the round goby (Neogobius melanostomus). Biol Invasions.

[32] Farwell M, Hughes G, Smith JL, Clelland E, Loeb SJ, Semeniuk C (2017). Differential female preference for individual components of a reproductive male round goby (Neogobius melanostomus) pheromone. J Great Lakes Res.

[33] Tierney KB, Kereliuk M, Katare YK, Scott AP, Loeb SJ, Zielinski B (2012). Invasive male round gobies (Neogobius melanostomus) release pheromones in their urine to attract females. Can J Fish Aquat Sci.

[34] Laframboise AJ, Katare Y, Scott AP, Zielinski BS (2011). The effect of elevated steroids released by reproductive male round gobies, Neogobius melanostomus, on olfactory responses in females. J Chem Ecol.

[35] Marentette JR, Gooderham KL, McMaster ME, Ng T, Parrott JL, Wilson JY (2010). Signatures of contamination in invasive round gobies (Neogobius melanostomus): a double strike for ecosystem health?. Ecotoxicol Environ Saf.

[36] Marentette JR, Balshine S (2012). Altered prey responses in round goby from contaminated sites. Ethology.

[37] McCallum ES, Charney RE, Marenette JR, Young JAM, Koops MA, Earn DJD (2014). Persistence of an invasive fish (Neogobius melanostomus) in a contaminated ecosystem. Biol Invasions.

[38] Goldstone JV, Hamdoun A, Cole BJ, Howard-Ashby M, Nebert DW, Scally M (2006). The chemical defensome: environmental sensing and response genes in the Strongylocentrotus purpuratus genome. Dev Biol.

[39] Ellis SM, MacIsaac HJ (2009). Salinity tolerance of Great Lakes invaders. Freshw Biol.

[40] Karsiotis SI, Pierce LR, Brown JE, Stepien CA (2012). Salinity tolerance of the invasive round goby: experimental implications for seawater ballast exchange and spread to north American estuaries. J Great Lakes Res.

[41] Lee KA, Klasing KC (2004). A role for immunology in invasion biology. Trends Ecol Evolution.

[42] David GM, Staentzel C, Schlumberger O, Perrot-Minnot M, Beisel J, Hardion L (2018). A minimalist macroparasite diversity in the round goby of the Upper Rhine reduced to an exotic acanthocephalan lineage. Parasitology.

[43] Jaenisch R, Bird A (2003). Epigenetic regulation of gene expression: How the genome integrates intrinsic and environmental signals. Nature Genetics.

[44] Zamudio N, Barau J, Teissandier A, Walter M, Borsos M, Servant N (2015). DNA methylation restrains transposons from adopting a chromatin signature permissive for meiotic recombination. Genes Dev.

[45] Choi J, Lyons DB, Kim Y, Moore JD, Zilberman D. DNA methylation and histone H1 cooperatively repress transposable elements and aberrant intragenic transcripts. bioRxiv 2019:527523. 10.1016/j.molcel.2019.10.011 31732458

[46] Feinberg AP, Irizarry RA (2010). Stochastic epigenetic variation as a driving force of development, evolutionary adaptation, and disease. Proc Natl Acad Sci.

[47] Herman JJ, Sultan SE (2016). DNA methylation mediates genetic variation for adaptive transgenerational plasticity. Proc R Soc B Biol Sci.

[48] Cortijo S, Wardenaar R, Colomé-Tatché M, Gilly A, Etcheverry M, Labadie K (2014). Mapping the epigenetic basis of complex traits. Science.

[49] Wellband KW, Heath DD (2017). Plasticity in gene transcription explains the differential performance of two invasive fish species. Evol Appl.

[50] Adrian-Kalchhauser I, Walser J, Schwaiger M, Burkhardt-Holm P (2018). RNA sequencing of early round goby embryos reveals that maternal experiences can shape the maternal RNA contribution in a wild vertebrate. BMC Evol Biol.

[51] Somerville Vincent, Schwaiger Michaela, Hirsch Philipp E., Walser Jean-Claude, Bussmann Karen, Weyrich Alexandra, Burkhardt-Holm Patricia, Adrian-Kalchhauser Irene (2019). DNA Methylation Patterns in the Round Goby Hypothalamus Support an On-The-Spot Decision Scenario for Territorial Behavior. Genes.

[52] Grimm SA, Shimbo T, Takaku M, Thomas JW, Auerbach S, Bennett BD (2019). DNA methylation in mice is influenced by genetics as well as sex and life experience. Nat Commun.

[53] Weyrich Alexandra, Benz Stephanie, Karl Stephan, Jeschek Marie, Jewgenow Katarina, Fickel Joerns (2016). Paternal heat exposure causes DNA methylation and gene expression changes ofStat3in Wild guinea pig sons. Ecology and Evolution.

[54] Margueron R, Reinberg D (2011). The Polycomb complex PRC2 and its mark in life. Nature.

[55] Gibbs DJ, Tedds HM, Labandera A, Bailey M, White MD, Hartman S (2018). Oxygen-dependent proteolysis regulates the stability of angiosperm polycomb repressive complex 2 subunit VERNALIZATION 2. Nat Commun.

[56] Martinez P, Vinas AM, Sanchez L, Diaz N, Ribas L, Piferrer F. Genetic architecture of sex determination in fish: applications to sex ratio control in aquaculture. Front Genet. 2014;5:340. 10.3389/fgene.2014.00340 PMC4179683 25324858

[57] Hardie DC, Hebert PD (2003). The nucleotypic effects of cellular DNA content in cartilaginous and ray-finned fishes. Genome.

[58] Hardie DC, Hebert PD (2004). Genome-size evolution in fishes. Can J Fish Aquat Sci.

[59] Gregory TR. Animal Genome Size Database 2019. Available from: URL: http://www.genomesize.com.

[60] Adrian-Kalchhauser I, Blomberg A, Larsson T, Musilova Z, Peart CR, Pippel M et al. Neogobius melanostomus isolate 20150602_KH_C, whole genome shotgun sequencing project, Genbank accession number VHKM00000000; 2019. Available from: URL: https://www.ncbi.nlm.nih.gov/nuccore/VHKM00000000.

[61] Wellband KW, Heath DD. Round goby liver transcriptome, NCBI SRA archive, BioProject accession: PRJNA321539. Available from: URL: https://www.ncbi.nlm.nih.gov/bioproject/PRJNA321539.

[62] Adrian-Kalchhauser I. Round goby RAD sequencing data, NCBI SRA Archive, BioProject Accession: PRJNA547536. Available from: https://www.ncbi.nlm.nih.gov/bioproject/PRJNA547536.

[63] Adrian-Kalchhauser I. Round goby RAD sequencing data, NCBI SRA archive, BioProject accession: PRJNA515617. Available from: URL: https://www.ncbi.nlm.nih.gov/bioproject/PRJNA515617.

[64] Adrian-Kalchhauser I. Round goby liver and brain ATACseq data, NCBI SRA archive, BioProject accession: PRJNA551348. Available from: URL: https://www.ncbi.nlm.nih.gov/bioproject/PRJNA551348.

[65] Adrian-Kalchhauser I. Round goby brain methylome, NCBI SRA Archive, BioProject Accession: PRJNA515617. Available from: https://www.ncbi.nlm.nih.gov/bioproject/PRJNA515617.

[66] Cortesi F, Musilova Z, Stieb SM, Hart NS, Siebeck UE, Malmstrom M (2015). Ancestral duplications and highly dynamic opsin gene evolution in percomorph fishes. Proc Natl Acad Sci.

[67] Liu D, Wang F, Lin J, Thompson A, Lu Y, Vo D (2019). The cone opsin repertoire of osteoglossomorph fishes: gene loss in mormyrid electric fish and a long wavelength-sensitive cone opsin that survived 3R. Mol Biol Evol.

[68] Yokoyama Shozo (2008). Evolution of Dim-Light and Color Vision Pigments. Annual Review of Genomics and Human Genetics.

[69] Michiels NK, Anthes N, Hart NS, Herler J, Meixner AJ, Schleifenbaum F (2008). Red fluorescence in reef fish: a novel signalling mechanism?. BMC Ecol.

[70] Niimura Y (2012). Olfactory receptor multigene family in vertebrates: from the viewpoint of evolutionary genomics. Curr Genomics.

[71] Niimura Y (2009). On the origin and evolution of vertebrate olfactory receptor genes: comparative genome analysis among 23 chordate species. Genome Biol Evolution.

[72] Nelson DR (2003). Comparison of P450s from human and fugu: 420 million years of vertebrate P450 evolution. Arch Biochem Biophys.

[73] Goldstone JV, McArthur AG, Kubota A, Zanette J, Parente T, Jönsson ME (2010). Identification and developmental expression of the full complement of cytochrome P450 genes in Zebrafish. BMC Genomics.

[74] Zhang J, Yao J, Wang R, Zhang Y, Liu S, Sun L (2014). The cytochrome P450 genes of channel catfish: their involvement in disease defense responses as revealed by meta-analysis of RNA-Seq data sets. Biochim Biophys Acta.

[75] Kirischian N, McArthur AG, Jesuthasan C, Krattenmacher B, Wilson JY (2011). Phylogenetic and functional analysis of the vertebrate cytochrome p450 2 family. J Mol Evol.

[76] Dejong CA, Wilson JY (2014). The cytochrome P450 superfamily complement (CYPome) in the annelid Capitella teleta. PLoS One.

[77] Luch Andreas, Baird William M. (2005). Metabolic Activation and Detoxification of Polycyclic Aromatic Hydrocarbons. The Carcinogenic Effects of Polycyclic Aromatic Hydrocarbons.

[78] Yan J, Cai Z (2010). Molecular evolution and functional divergence of the cytochrome P450 3 (CYP3) family in Actinopterygii (ray-finned fish). PLoS One.

[79] Yokoyama C, Yabuki T, Inoue H, Tone Y, Hara S, Hatae T (1996). Human gene encoding prostacyclin synthase (PTGIS): genomic organization, chromosomal localization, and promoter activity. Genomics.

[80] Li Y, Chiang C, Yeh H, Hsu P, Whitby FG, Wang L (2008). Structures of prostacyclin synthase and its complexes with substrate analog and inhibitor reveal a ligand-specific heme conformation change. J Biol Chem.

[81] Finn RN, Cerdà J (2011). Aquaporin evolution in fishes. Front Physiol.

[82] Finn RN, Chauvigné F, Hlidberg JB, Cutler CP, Cerdà J (2014). The lineage-specific evolution of aquaporin gene clusters facilitated tetrapod terrestrial adaptation. PLoS One.

[83] Loh YH, Christoffels A, Brenner S, Hunziker W, Venkatesh B (2004). Extensive expansion of the claudin gene family in the teleost fish, Fugu rubripes. Genome Research.

[84] Hwang P, Chou M (2013). Zebrafish as an animal model to study ion homeostasis. Pflugers Arch - Eur J Physiol.

[85] Ronkin D, Seroussi E, Nitzan T, Doron-Faigenboim A, Cnaani A (2015). Intestinal transcriptome analysis revealed differential salinity adaptation between two tilapiine species. Comp Biochem Physiol Part D Genomics Proteomics.

[86] Rim JS, Atta MG, Dahl SC, Berry GT, Handler JS, Kwon HM (1998). Transcription of the sodium/myo-inositol cotransporter gene is regulated by multiple tonicity-responsive enhancers spread over 50 kilobase pairs in the 5 ‘-flanking region. J Biol Chem.

[87] Wang X, Kültz D (2017). Osmolality/salinity-responsive enhancers (OSREs) control induction of osmoprotective genes in euryhaline fish. Proc Natl Acad Sci.

[88] Sacchi R, Gardell AM, Chang N, Kültz D (2014). Osmotic regulation and tissue localization of the myo-inositol biosynthesis pathway in tilapia (Oreochromis mossambicus) larvae. J Exp Zool A Ecol Genet Physiol.

[89] Sacchi R, Li J, Villarreal F, Gardell AM, Kültz D (2013). Salinity-induced regulation of the &lt;em&gt;myo&lt;/em&gt;-inositol biosynthesis pathway in tilapia gill epithelium. J Exp Biol.

[90] Flajnik MF (2018). A cold-blooded view of adaptive immunity. Nat Rev Immunol.

[91] Grimholt U, Tsukamoto K, Azuma T, Leong J, Koop BF, Dijkstra JM (2015). A comprehensive analysis of teleost MHC class I sequences. BMC Evol Biol.

[92] McConnell SC, Hernandez KM, Wcisel DJ, Kettleborough RN, Stemple DL, Yoder JA (2016). Alternative haplotypes of antigen processing genes in zebrafish diverged early in vertebrate evolution. Proc Natl Acad Sci.

[93] Mashoof S, Criscitiello MF (2016). Fish Immunoglobulins. Biology (Basel).

[94] Riera Romo M, Perez-Martinez D, Castillo FC (2016). Innate immunity in vertebrates: an overview. Immunology.

[95] Guo H, Callaway JB, Ting JP (2015). Inflammasomes: mechanism of action, role in disease, and therapeutics. Nat Med.

[96] Nie L, Cai S, Shao J, Chen J (2018). Toll-like receptors, associated biological roles, and signaling networks in non-mammals. Front Immunol.

[97] Zhang J, Liu S, Rajendran KV, Sun L, Zhang Y, Sun F (2013). Pathogen recognition receptors in channel catfish: III phylogeny and expression analysis of toll-like receptors. Dev Comp Immunol.

[98] Solbakken MH, Tørresen OK, Nederbragt AJ, Seppola M, Gregers TF, Jakobsen KS (2016). Evolutionary redesign of the Atlantic cod (Gadus morhua L.) Toll-like receptor repertoire by gene losses and expansions. Sci Rep.

[99] Solbakken Monica Hongrø, Voje Kjetil Lysne, Jakobsen Kjetill Sigurd, Jentoft Sissel (2017). Linking species habitat and past palaeoclimatic events to evolution of the teleost innate immune system. Proceedings of the Royal Society B: Biological Sciences.

[100] Lupfer C, Kanneganti T (2013). Unsolved mysteries in NLR biology. Front Immunol.

[101] Laing Kerry J, Purcell Maureen K, Winton James R, Hansen John D (2008). A genomic view of the NOD-like receptor family in teleost fish: identification of a novel NLR subfamily in zebrafish. BMC Evolutionary Biology.

[102] Howe K, Schiffer PH, Zielinski J, Wiehe T, Laird GK, Marioni JC (2016). Structure and evolutionary history of a large family of NLR proteins in the zebrafish. Open Biol.

[103] Tørresen OK, Brieuc MSO, Solbakken MH, Sørhus E, Nederbragt AJ, Jakobsen KS (2018). Genomic architecture of haddock (Melanogrammus aeglefinus) shows expansions of innate immune genes and short tandem repeats. BMC Genomics.

[104] Li J, Chu Q, Xu T (2016). A genome-wide survey of expansive NLR-C subfamily in miiuy croaker and characterization of the NLR-B30.2 genes. Dev Comp Immunol.

[105] Xu T, Xu G, Che R, Wang R, Wang Y, Li J (2016). The genome of the miiuy croaker reveals well-developed innate immune and sensory systems. Sci Rep.

[106] Rajendran KV, Zhang J, Liu S, Kucuktas H, Wang X, Liu H (2012). Pathogen recognition receptors in channel catfish: I. identification, phylogeny and expression of NOD-like receptors. Dev Comp Immunol.

[107] Li J, Gao K, Shao T, Fan D, Hu C, Sun C (2018). Characterization of an NLRP1 Inflammasome from zebrafish reveals a unique sequential activation mechanism underlying inflammatory caspases in ancient vertebrates. J Immunol.

[108] Kuri P, Schieber NL, Thumberger T, Wittbrodt J, Schwab Y, Leptin M (2017). Dynamics of in vivo ASC speck formation. J Cell Biol.

[109] Schwartz YB, Pirrotta V (2013). A new world of Polycombs: Unexpected partnerships and emerging functions. Nat Rev Genet.

[110] Mu W, Starmer J, Shibata Y (2017). Della Yee, Magnuson T. EZH1 in germ cells safeguards the function of PRC2 during spermatogenesis. Dev Biol.

[111] Xu J, Shao Z, Li D, Xie H, Kim W, Huang J (2015). Developmental control of polycomb subunit composition by GATA factors mediates a switch to non-canonical functions. Mol Cell.

[112] San B, Chrispijn ND, Wittkopp N, van Heeringen SJ, Lagendijk AK, Aben M (2016). Normal formation of a vertebrate body plan and loss of tissue maintenance in the absence of ezh2. Sci Rep.

[113] Völkel P, Bary A, Raby L, Chapart A, Dupret B, Le Bourhis X (2019). Ezh1 arises from Ezh2 gene duplication but its function is not required for zebrafish development. Sci Rep.

[114] Jeltsch A, Jurkowska RZ (2014). New concepts in DNA methylation. Trends Biochem Sci.

[115] Ponger L, Li W (2005). Evolutionary diversification of DNA Methyltransferases in eukaryotic genomes. Mol Biol Evol.

[116] Wang F, Yan L, Shi H, Liu X, Zheng Q, Sun L (2018). Genome-wide identification, evolution of DNA methyltransferases and their expression during gonadal development in Nile tilapia. Comp Biochem Physiol B: Biochem Mol Biol.

[117] Stapley J, Santure AW, Dennis SR (2015). Transposable elements as agents of rapid adaptation may explain the genetic paradox of invasive species. Mol Ecol.

[118] Pysek P, Skalova H, Cuda J, Guo W, Suda J, Dolezal J (2018). Small genome separates native and invasive populations in an ecologically important cosmopolitan grass. Ecology.

[119] Pezold FL (1984). Evidence for multiple sex-chromosomes in the fresh-water goby Gobionellus Shufeldti (Pisces, Gobiidae). Copeia.

[120] Jaillon O, Aury JM, Brunet F, Petit JL, Stange-Thomann N, Mauceli E (2004). Genome duplication in the teleost fish Tetraodon nigroviridis reveals the early vertebrate proto-karyotype. Nature.

[121] Pauli A, Valen E, Lin MF, Garber M, Vastenhouw NL, Levin JZ (2012). Systematic identification of long noncoding RNAs expressed during zebrafish embryogenesis. Genome Res.

[122] Bitton P, Christmann SAY, Santon M, Harant UK, Michiels NK. Visual modelling validates prey detection by means of diurnal active photolocation in a small cryptobenthic fish. bioRxiv 2018:338640. 10.1038/s41598-019-44529-0 PMC6542814 31147614

[123] Carleton KL, Dalton BE, Escobar-Camacho D, Nandamuri SP (2016). Proximate and ultimate causes of variable visual sensitivities: insights from cichlid fish radiations. Genesis.

[124] Marshall Justin, Johnsen Sonke (2017). Fluorescence as a means of colour signal enhancement. Philosophical Transactions of the Royal Society B: Biological Sciences.

[125] Olsson KH, Johansson S, Blom E, Lindström K, Svensson O, Nilsson Sköld H (2017). Dark eyes in female sand gobies indicate readiness to spawn. PLoS One.

[126] Anthes N, Theobald J, Gerlach T, Meadows MG, Michiels NK. Diversity and ecological correlates of red fluorescence in marine fishes. Front Ecol Evol. 2016;4:216.

[127] Vélez-Espino LA, Koops MA, Balshine S (2010). Invasion dynamics of round goby (Neogobius melanostomus) in Hamilton Harbour, Lake Ontario. Biological Invasions.

[128] Young JAM, Marentette JR, Gross C, McDonald JI, Verma A, Marsh-Rollo SE (2010). Demography and substrate affinity of the round goby (Neogobius melanostomus) in Hamilton Harbour. J Great Lakes Res.

[129] Behrens JW, van Deurs M, Christensen EAF (2017). Evaluating dispersal potential of an invasive fish by the use of aerobic scope and osmoregulation capacity. PLoS One.

[130] Hirsch PE, Adrian-Kalchhauser I, Flämig S, N’Guyen A, Defila R, Di Giulio A (2016). A tough egg to crack: recreational boats as vectors for invasive goby eggs and transdisciplinary management approaches. Ecol Evolution.

[131] Miladi H, Elabed H, Ben Slama R, Rhim A, Bakhrouf A (2017). Molecular analysis of the role of osmolyte transporters opuCA and betL in Listeria monocytogenes after cold and freezing stress. Arch Microbiol.

[132] Vigoder FM, Parker DJ, Cook N, Tournière O, Sneddon T, Ritchie MG (2016). Inducing cold-sensitivity in the Frigophilic Fly Drosophila montana by RNAi. PLoS One.

[133] Reis Marta I. R., do Vale Ana, Pereira Pedro J. B., Azevedo Jorge E., dos Santos Nuno M. S. (2012). Caspase-1 and IL-1β Processing in a Teleost Fish. PLoS ONE.

[134] Vojtech LN, Scharping N, Woodson JC, Hansen JD (2012). Roles of inflammatory caspases during processing of zebrafish interleukin-1β in Francisella noatunensis infection. Infect Immun.

[135] Richter K, Sagawe S, Hecker A, Küllmar M, Askevold I, Damm J (2018). C-reactive protein stimulates nicotinic acetylcholine receptors to control ATP-mediated monocytic inflammasome activation. Front Immunol.

[136] Cao R, Zhang Y (2004). SUZ12 is required for both the histone methyltransferase activity and the silencing function of the EED-EZH2 complex. Mol Cell.

[137] Ciferri C, Lander GC, Maiolica A, Herzog F, Aebersold R, Nogales E (2012). Molecular architecture of human polycomb repressive complex 2. Elife.

[138] Chittock EC, Latwiel S, Miller TCR, Müller CW (2017). Molecular architecture of polycomb repressive complexes. Biochem Soc Trans.

[139] Cao Q, Wang X, Zhao M, Yang R, Malik R, Qiao Y (2014). The central role of EED in the orchestration of polycomb group complexes. Nat Commun.

[140] Liu X, Yang J, Wu N, Song R, Zhu H (2015). Evolution and coevolution of PRC2 genes in vertebrates and mammals. Adv Protein Chem Struct Biol.

[141] Davidovich C, Cech TR (2015). The recruitment of chromatin modifiers by long noncoding RNAs: lessons from PRC2. RNA.

[142] Cross S, Kovarik P, Schmidtke J, Bird A (1991). Non-methylated islands in fish genomes are GC-poor. Nucleic Acids Res.

[143] Jiang N, Wang L, Chen J, Wang L, Leach L, Luo Z (2014). Conserved and divergent patterns of DNA methylation in higher vertebrates. Genome Biol Evolution.

[144] Han L, Zhao Z. Comparative analysis of CpG islands in four fish genomes. Comp Funct Genomics. 2008;565631 10.1155/2008/565631 PMC2375969 18483567

[145] Huska Matthew, Vingron Martin (2016). Improved Prediction of Non-methylated Islands in Vertebrates Highlights Different Characteristic Sequence Patterns. PLOS Computational Biology.

[146] McGaughey DM, Abaan HO, Miller RM, Kropp PA, Brody LC (2014). Genomics of CpG methylation in developing and developed zebrafish. G3 (Bethesda).

[147] Skvortsova K, Tarbashevich K, Stehling M, Lister R, Irimia M, Raz E (2019). Retention of paternal DNA methylome in the developing zebrafish germline. Nat Commun.

[148] Potok ME, Nix DA, Parnell TJ, Cairns BR (2013). Reprogramming the maternal zebrafish genome after fertilization to match the paternal methylation pattern. Cell.

[149] Fellous Alexandre, Labed-Veydert Tiphaine, Locrel Mélodie, Voisin Anne-Sophie, Earley Ryan L., Silvestre Frederic (2018). DNA methylation in adults and during development of the self-fertilizing mangrove rivulus, Kryptolebias marmoratus. Ecology and Evolution.

[150] Wang X, Bhandari RK (2019). DNA methylation dynamics during epigenetic reprogramming of medaka embryo. Epigenetics.

[151] Campos C, Valente LM, Fernandes JM (2012). Molecular evolution of zebrafish dnmt3 genes and thermal plasticity of their expression during embryonic development. Gene.

[152] Takayama K, Shimoda N, Takanaga S, Hozumi S, Kikuchi Y (2014). Expression patterns of dnmt3aa, dnmt3ab, and dnmt4 during development and fin regeneration in zebrafish. Gene Expr Patterns.

[153] Firmino J, Carballo C, Armesto P, Campinho MA, Power DM, Manchado M (2017). Phylogeny, expression patterns and regulation of DNA methyltransferases in early development of the flatfish, *Solea senegalensis*. BMC Developmental Biol.

[154] Wood RK, Crowley E, Martyniuk CJ (2016). Developmental profiles and expression of the DNA methyltransferase genes in the fathead minnow (Pimephales promelas) following exposure to di-2-ethylhexyl phthalate. Fish Physiol Biochem.

[155] Berthelot C, Brunet F, Chalopin D, Juanchich A, Bernard M, Noël B (2014). The rainbow trout genome provides novel insights into evolution after whole-genome duplication in vertebrates. Nat Commun.

[156] Lien S, Koop BF, Sandve SR, Miller JR, Kent MP, Nome T (2016). The Atlantic salmon genome provides insights into rediploidization. Nature.

[157] Kim B, Amores A, Kang S, Ahn D, Kim J, Kim I (2019). Antarctic blackfin icefish genome reveals adaptations to extreme environments. Nat Ecol Evol.

[158] Mu Y, Huo J, Guan Y, Fan D, Xiao X, Wei J (2018). An improved genome assembly for Larimichthys crocea reveals hepcidin gene expansion with diversified regulation and function. Commun Biol.

[159] Liu Z, Liu S, Yao J, Bao L, Zhang J, Li Y, et al. The channel catfish genome sequence provides insights into the evolution of scale formation in teleosts. Nat Commun. 2016;7 10.1038/ncomms11757 PMC4895719 27249958

[160] Wu C, Di Zhang, Kan M, Lv Z, Zhu A, Su Y et al. The draft genome of the large yellow croaker reveals well-developed innate immunity. Nat Commun. 2014;5:5227. 10.1038/ncomms6227 PMC4263168 25407894

[161] El-Brolosy MA, Kontarakis Z, Rossi A, Kuenne C, Günther S, Fukuda N (2019). Genetic compensation triggered by mutant mRNA degradation. Nature.

[162] Kondrashov FA (2012). Gene duplication as a mechanism of genomic adaptation to a changing environment. Proc R Soc B Biol Sci.

[163] Wu N, Zhang S, Li X, Cao Y, Liu X, Wang Q (2019). Fall webworm genomes yield insights into rapid adaptation of invasive species. Nat Ecol Evol.

[164] Makino T, Kawata M (2019). Invasive invertebrates associated with highly duplicated gene content. Mol Ecol.

[165] Roche K, Janáč M, Šlapanský L, Mikl L, Kopeček L, Jurajda P (2015). A newly established round goby (Neogobius melanostomus ) population in the upper stretch of the river Elbe. Knowl Manag Aquat Ecosyst.

[166] Patzner RA, VanTassel J.L., Kovačić M, Kapoor BG, editors. The biology of gobies. Enfield: Science Publishers; 2011.

[167] Xing J, Zhou X, Tang X, Sheng X, Zhan W (2017). Characterization of toll-like receptor 22 in turbot (Scophthalmus maximus). Fish Shellfish Immunol.

[168] Paria A, Makesh M, Chaudhari A, Purushothaman CS, Rajendran KV (2018). Toll-like receptor (TLR) 22, a non-mammalian TLR in Asian seabass, Lates calcarifer: characterisation, ontogeny and inductive expression upon exposure with bacteria and ligands. Dev Comp Immunol.

[169] Qi Z, Wang S, Zhu X, Yang Y, Han P, Zhang Q (2018). Molecular characterization of three toll-like receptors (TLR21, TLR22, and TLR25) from a primitive ray-finned fish Dabry’s sturgeon (Acipenser dabryanus). Fish Shellfish Immunol.

[170] White RJ, Collins JE, Sealy IM, Wali N, Dooley CM, Digby Z, et al. A high-resolution mRNA expression time course of embryonic development in zebrafish. Elife. 2017;6:e30860. 10.7554/eLife.30860 PMC5690287 29144233

[171] Zhang L, Gao Z, Yu L, Zhang B, Wang J, Zhou J (2018). Nucleotide-binding and oligomerization domain (NOD)-like receptors in teleost fish: current knowledge and future perspectives. J Fish Dis.

[172] Nowoshilow S, Schloissnig S, Fei J, Dahl A, Pang AWC, Pippel M (2018). The axolotl genome and the evolution of key tissue formation regulators. Nature.

[173] Grohme MA, Schloissnig S, Rozanski A, Pippel M, Young GR, Winkler S (2018). The genome of Schmidtea mediterranea and the evolution of core cellular mechanisms. Nature.

[174] Chin C, Alexander DH, Marks P, Klammer AA, Drake J, Heiner C (2013). Nonhybrid, finished microbial genome assemblies from long-read SMRT sequencing data. Nature Methods.

[175] Cantarel BL, Korf I, Robb SMC, Parra G, Ross E, Moore B (2008). MAKER: an easy-to-use annotation pipeline designed for emerging model organism genomes. Genome Res.

[176] Campbell MS, Holt C, Moore B, Yandell M (2014). Genome annotation and Curation using MAKER and MAKER-P. Curr Protoc Bioinformatics.

[177] Zerbino DR, Achuthan P, Akanni W, Amode MR, Barrell D, Bhai J (2018). Ensembl 2018. Nucleic Acids Res.

[178] The UniProt Consortium (2019). UniProt: a worldwide hub of protein knowledge. Nucleic Acids Res.

[179] Korf Ian (2004). BMC Bioinformatics.

[180] Stanke M, Diekhans M, Baertsch R, Haussler D (2008). Using native and syntenically mapped cDNA alignments to improve de novo gene finding. Bioinformatics.

[181] Smit A, Hubley R, Green P. RepeatMasker Open-4.0; 2013-2015.

[182] Bao W, Kojima KK, Kohany O (2015). Repbase update, a database of repetitive elements in eukaryotic genomes. Mob DNA.

[183] Waterhouse RM, Seppey M, Simão FA, Manni M, Ioannidis P, Klioutchnikov G (2017). BUSCO applications from quality assessments to gene prediction and phylogenomics. Mol Biol Evol.

[184] Kriventseva EV, Zdobnov EM, Simão FA, Ioannidis P, Waterhouse RM (2015). BUSCO: assessing genome assembly and annotation completeness with single-copy orthologs. Bioinformatics.

[185] Dunn NA, Unni D, Buels R, Sargent L, Diesch C, Lewis SE et al. GMOD/Apollo: 2.2.0 JB#1.15.4-release.

[186] Lee E, Helt GA, Reese JT, Munoz-Torres MC, Childers CP, Buels RM (2013). Web Apollo: a web-based genomic annotation editing platform. Genome Biol.

[187] Baird NA, Etter PD, Atwood TS, Currey MC, Shiver AL, Lewis ZA (2008). Rapid SNP discovery and genetic mapping using sequenced RAD markers. PLoS One.

[188] Roesti M, Hendry AP, Salzburger W, Berner D (2012). Genome divergence during evolutionary diversification as revealed in replicate lake-stream stickleback population pairs. Mol Ecol.

[189] Roesti M, Kueng B, Moser D, Berner D (2015). The genomics of ecological vicariance in threespine stickleback fish. Nat Commun.

[190] Hohenlohe PA, Bassham S, Etter PD, Stiffler N, Johnson EA, Cresko WA (2010). Population genomics of parallel adaptation in Threespine stickleback using sequenced RAD tags. PLoS Genet.

[191] Rochette NC, Catchen JM (2017). Deriving genotypes from RAD-seq short-read data using stacks. Nat Protoc.

[192] Ocalewicz K, Sapota M (2011). Cytogenetic characteristics of the round goby Neogobius melanostomus (Pallas, 1814) (Teleostei: Gobiidae: Benthophilinae). Mar Biol Res.

[193] Kearse M, Moir R, Wilson A, Stones-Havas S, Cheung M, Sturrock S (2012). Geneious basic: an integrated and extendable desktop software platform for the organization and analysis of sequence data. Bioinformatics.

[194] Ward MN, Churcher AM, Dick KJ, Laver CRJ, Owens GL, Polack MD (2008). The molecular basis of color vision in colorful fish: four long wave-sensitive (LWS) opsins in guppies (Poecilia reticulata) are defined by amino acid substitutions at key functional sites. BMC Evol Biol.

[195] Rennison DJ, Owens GL, Taylor JS (2012). Opsin gene duplication and divergence in ray-finned fish. Mol Phylogenet Evol.

[196] Lin J, Wang F, Li W, Wang T. The rises and falls of opsin genes in 59 ray-finned fish genomes and their implications for environmental adaptation. Sci Rep. 2017;7:15568. 10.1038/s41598-017-15868-7 PMC5686071 29138475

[197] Register EA, Yokoyama R, Yokoyama S (1994). Multiple origins of the green-sensitive opsin genes in fish. J Mol Evol.

[198] Katoh K, Kuma K, Toh H, Miyata T (2005). MAFFT version 5: improvement in accuracy of multiple sequence alignment. Nucleic Acids Res.

[199] Darriba D, Taboada GL, Doallo R, Posada D (2012). jModelTest 2: more models, new heuristics and parallel computing. Nat Methods.

[200] Guindon S, Gascuel O (2003). A simple, fast, and accurate algorithm to estimate large phylogenies by maximum likelihood. Syst Biol.

[201] Ronquist F, Huelsenbeck JP (2003). MrBayes 3: Bayesian phylogenetic inference under mixed models. Bioinformatics.

[202] Hajkova P, Jeffries SJ, Lee C, Miller N, Jackson SP, Surani MA (2010). Genome-wide reprogramming in the mouse germ line entails the base excision repair pathway. Science.

[203] Brawand D, Wagner CE, Li YI, Malinsky M, Keller I, Fan S (2014). The genomic substrate for adaptive radiation in African cichlid fish. Nature.

[204] Peichel CL, Sullivan ST, Liachko I, White MA (2017). Improvement of the Threespine stickleback genome using a hi-C-based proximity-guided assembly. J Hered.

[205] Haas BJ, Papanicolaou A, Yassour M, Grabherr M, Blood PD, Bowden J (2013). De novo transcript sequence reconstruction from RNA-seq using the trinity platform for reference generation and analysis. Nat Protoc.

[206] Wheeler TJ, Eddy SR (2013). Nhmmer: DNA homology search with profile HMMs. Bioinformatics.

[207] Katoh K, Rozewicki J, Yamada KD (2019). MAFFT online service: multiple sequence alignment, interactive sequence choice and visualization. Brief Bioinform.

[208] Trifinopoulos J, Nguyen L, von Haeseler A, Minh BQ (2016). W-IQ-TREE: a fast online phylogenetic tool for maximum likelihood analysis. Nucleic Acids Res.

[209] Hoang DT, Chernomor O, von Haeseler A, Minh BQ, Le Vinh S (2018). UFBoot2: improving the ultrafast bootstrap approximation. Mol Biol Evol.

[210] Altschul S (1990). Basic local alignment search tool. J Mol Biol.

[211] Buels R, Yao E, Diesh CM, Hayes RD, Munoz-Torres M, Helt G (2016). JBrowse: a dynamic web platform for genome visualization and analysis. Genome Biol.

[212] Finn RD, Mistry J, Tate J, Coggill P, Heger A, Pollington JE (2010). The Pfam protein families database. Nucleic Acids Res.

[213] Sigrist CJA, Cerutti L, de Castro E, Langendijk-Genevaux PS, Bulliard V, Bairoch A (2010). PROSITE, a protein domain database for functional characterization and annotation. Nucleic Acids Res.

[214] Sievers F, Wilm A, Dineen D, Gibson TJ, Karplus K, Li W (2011). Fast, scalable generation of high-quality protein multiple sequence alignments using Clustal Omega. Mol Syst Biol.

[215] Maddison WP, Maddison MP. Mesquite: a modular system for evolutionary analysis. 2016; version 3.10. Available from: URL: http://mesquiteproject.org.

[216] Stamatakis A (2014). RAxML version 8: a tool for phylogenetic analysis and post-analysis of large phylogenies. Bioinformatics.

[217] Rambaut A. Figtree v1.4.3: Tree figure drawing tool. 2016. Available from: URL: http://tree.bio.ed.ac.uk/software/figtree/.

[218] O’Leary NA, Wright MW, Brister JR, Ciufo S, Haddad D, McVeigh R (2016). Reference sequence (RefSeq) database at NCBI: current status, taxonomic expansion, and functional annotation. Nucleic Acids Res.

[219] Guindon S, Dufayard J, Lefort V, Anisimova M, Hordijk W, Gascuel O (2010). New algorithms and methods to estimate maximum-likelihood phylogenies: assessing the performance of PhyML 3.0. Syst Biol.

[220] Camacho C, Coulouris G, Avagyan V, Ma N, Papadopoulos J, Bealer K (2009). BLAST+: architecture and applications. BMC Bioinformatics.

[221] Quinlan AR, Hall IM (2010). BEDTools: a flexible suite of utilities for comparing genomic features. Bioinformatics.

[222] Edgar RC (2004). MUSCLE: a multiple sequence alignment method with reduced time and space complexity. BMC Bioinformatics.

[223] Kumar Sudhir, Stecher Glen, Tamura Koichiro (2016). MEGA7: Molecular Evolutionary Genetics Analysis Version 7.0 for Bigger Datasets. Molecular Biology and Evolution.

[224] Darriba D, Taboada GL, Doallo R, Posada D (2011). ProtTest 3: fast selection of best-fit models of protein evolution. Bioinformatics.

[225] Stamatakis A (2006). RAxML-VI-HPC: maximum likelihood-based phylogenetic analyses with thousands of taxa and mixed models. Bioinformatics.

[226] Eddy Sean R. (2011). Accelerated Profile HMM Searches. PLoS Computational Biology.

[227] Katoh K, Standley DM (2013). MAFFT multiple sequence alignment software version 7: improvements in performance and usability. Mol Biol Evol.

[228] Wang P, Moore BM, Panchy NL, Meng F, Lehti-Shiu MD, Shiu S (2018). Factors influencing gene family size variation among related species in a plant family, Solanaceae. Genome Biol Evol.

[229] Crooks GE, Hon G, Chandonia J, Brenner SE (2004). WebLogo: a sequence logo generator. Genome Res.

[230] Olson SA (2002). EMBOSS opens up sequence analysis. European molecular biology open software suite. Brief Bioinform.

[231] Edwards JR, Yarychkivska O, Boulard M, Bestor TH (2017). DNA methylation and DNA methyltransferases. Epigenetics Chromatin.

[232] Ranwez V, Harispe S, Delsuc F, Douzery EJP (2011). MACSE: multiple alignment of coding SEquences accounting for frameshifts and stop codons. PLoS One.

[233] Lanfear R, Frandsen PB, Wright AM, Senfeld T, Calcott B (2017). PartitionFinder 2: new methods for selecting partitioned models of evolution for molecular and morphological phylogenetic analyses. Mol Biol Evol.

[234] Huelsenbeck JP, Ronquist F (2001). MRBAYES: Bayesian inference of phylogenetic trees. Bioinformatics.

[235] Smit A, Hubley R, Green P. RepeatModeler Open-1.0 2008-2015. Available from: URL: http://www.repeatmasker.org.

[236] Benson G (1999). Tandem repeats finder: a program to analyze DNA sequences. Nucleic Acids Res.

[237] Xu Z, Wang H (2007). LTR_FINDER: An efficient tool for the prediction of full-length LTR retrotransposons. Nucleic Acids Res.

[238] Ellinghaus D, Kurtz S, Willhoeft U (2008). LTRharvest, an efficient and flexible software for de novo detection of LTR retrotransposons. BMC Bioinformatics.

[239] Steinbiss S, Willhoeft U, Gremme G, Kurtz S (2009). Fine-grained annotation and classification of de novo predicted LTR retrotransposons. Nucleic Acids Res.

[240] Adrian-Kalchhauser I, Larsson T, Töpel M, Alm Rosenblad M. Round goby Neogobius melanostomus genome annotation: Zenodo; 2019. Available from: URL: 10.5281/zenodo.3561919.

