# Supplementary material for: The round goby genome provides insights into mechanisms that may facilitate biological invasions
Source: BMC Biol. 2020 Jan 28;18:11. doi: 10.1186/s12915-019-0731-8 (PMC6988351; doi:10.1186/s12915-019-0731-8)
Supplement: Supplementary file 3 — Figure S2. Phylogenetic tree of opsins constructed with individual exons. [file 12915_2019_731_MOESM3_ESM.pdf]

Supplemental_Fig_S2

The round goby genome

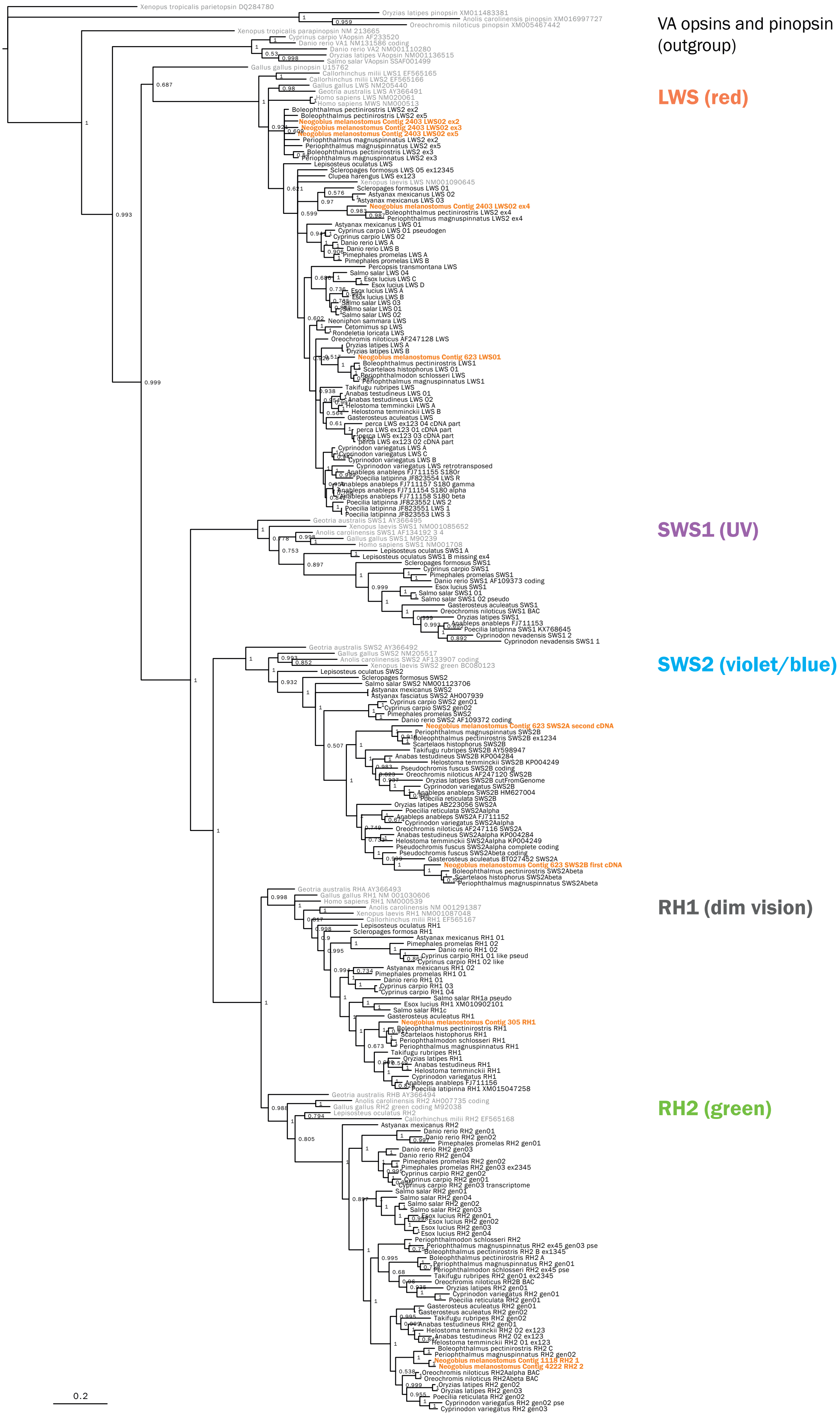

Phylogenetic tree of vertebrate opsin protein sequences, using single exons. Maximum-likelihood phylogenetic tree with VA opsins and pinopbsins as outgroup. Round goby is indicated in orange. Non-teleost species and the outgroup (VA opsins and pinopsins) are indicated in grey.
