# Supplementary material for: The round goby genome provides insights into mechanisms that may facilitate biological invasions
Source: BMC Biol. 2020 Jan 28;18:11. doi: 10.1186/s12915-019-0731-8 (PMC6988351; doi:10.1186/s12915-019-0731-8)

Supplemental_Fig_S3
The round goby genome

Maximum-likelihood phylogenetic tree of percomorph olfactory receptor protein sequences.

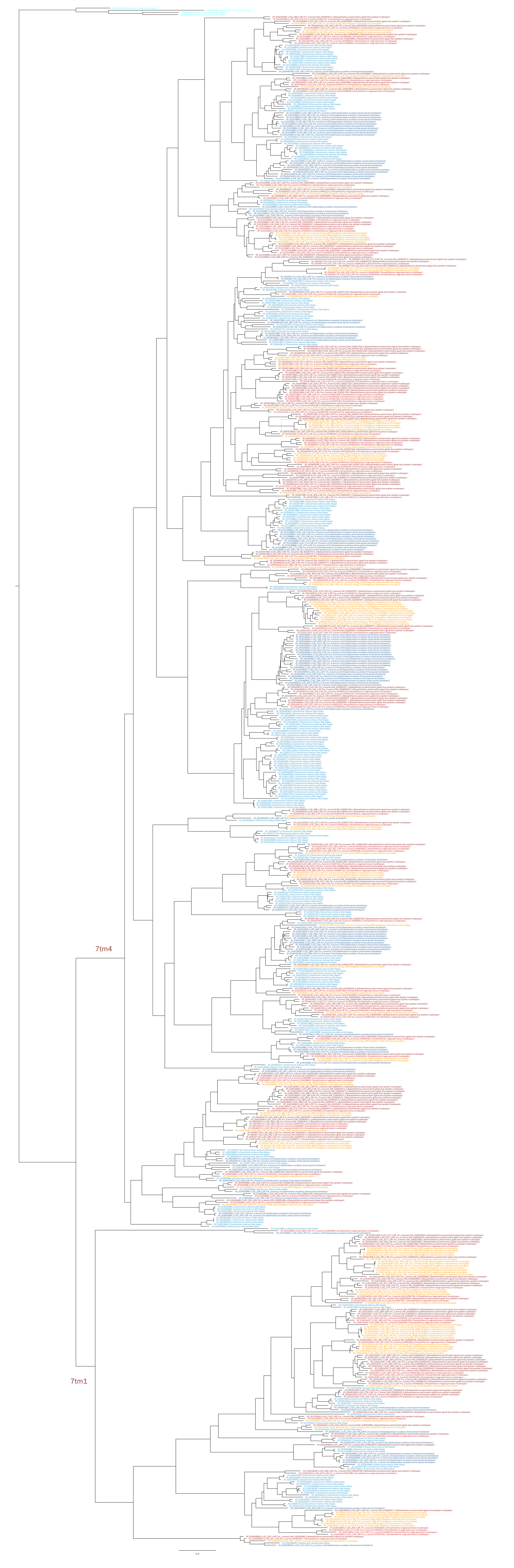

Supplement: Supplementary file 4 — Figure S3. Hylogenetic tree of olfactory receptors, with the brances that are depicted collapsed in Fig. 3 expanded. [file 12915_2019_731_MOESM4_ESM.pdf]
