# Supplementary figures and images for: The round goby genome provides insights into mechanisms that may facilitate biological invasions
Source: BMC Biol. 2020 Jan 28;18:11. doi: 10.1186/s12915-019-0731-8 (PMC6988351; doi:10.1186/s12915-019-0731-8)

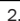

Supplement: Supplementary file 6 — Figure S4. Phylogenetic tree of claudins. [file 12915_2019_731_MOESM6_ESM.pdf]

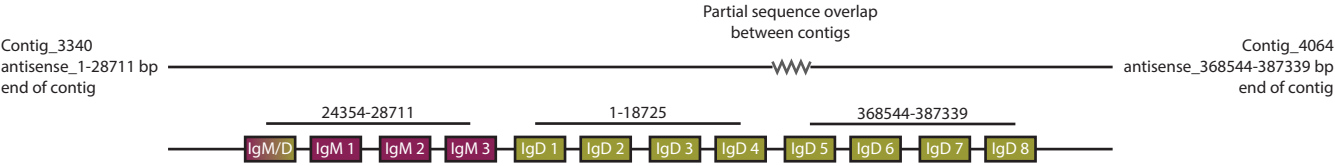

*The Ig locus spans two contigs and contains IgM and IgD domains.*

Supplement: Supplementary file 16 — Figure S9. Schematic of the immunoglobulin locus. [file 12915_2019_731_MOESM16_ESM.pdf]
