# Supplementary material for: The round goby genome provides insights into mechanisms that may facilitate biological invasions
Source: BMC Biol. 2020 Jan 28;18:11. doi: 10.1186/s12915-019-0731-8 (PMC6988351; doi:10.1186/s12915-019-0731-8)
Supplement: Supplementary file 9 — Figure S7. Phylogenetic trees of genes involved in myo-inositol production and accumulation. [file 12915_2019_731_MOESM9_ESM.pdf]

Supplemental_Fig_S7
The round goby genome

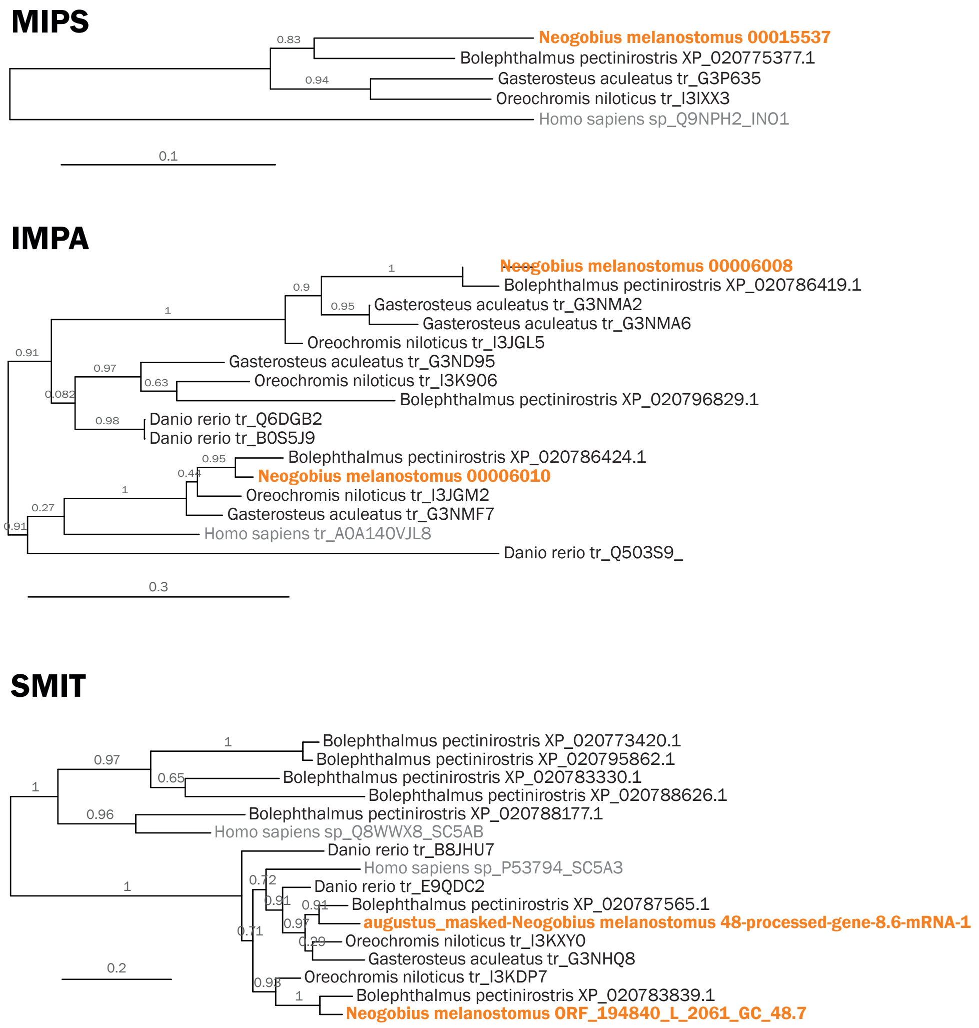


*Phylogenetic tree of vertebrate genes promoting osmolyte production. Maximum-likelihood tree with 100 bootstraps of round goby (Neogobius melanostomus, orange) in relation to great blue-spotted mudskipper (Boleophthalmus pectinirostris), stickleback (Gasterosteus aculeatus), nile tilapia (Oreochromis niloticus), zebrafish (Danio rerio), and human (Homo sapiens, grey)*
