# Supplementary material for: The round goby genome provides insights into mechanisms that may facilitate biological invasions
Source: BMC Biol. 2020 Jan 28;18:11. doi: 10.1186/s12915-019-0731-8 (PMC6988351; doi:10.1186/s12915-019-0731-8)
Supplement: Supplementary file 14 — Figure S8. Phylogenetic tree of TAP genes. [file 12915_2019_731_MOESM14_ESM.pdf]

Supplemental_Fig_S8
The round goby genome

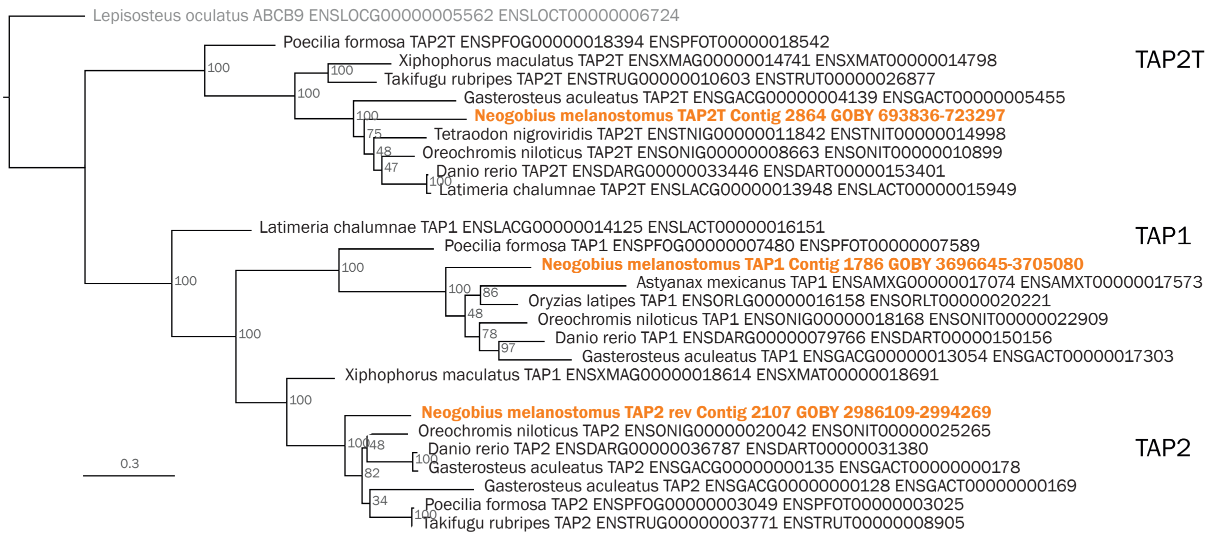

Phylogenetic tree of fish TAP genes. Maximum-likelihood tree with 500 bootstraps. Round goby (Neogobius melanostomus) is labeled orange. Outgroup: Lepistosteus oculatus ABC transporter.
