# Supplementary material for: The round goby genome provides insights into mechanisms that may facilitate biological invasions
Source: BMC Biol. 2020 Jan 28;18:11. doi: 10.1186/s12915-019-0731-8 (PMC6988351; doi:10.1186/s12915-019-0731-8)
Supplement: Supplementary file 18 — Figure S10. Phylogenetic tree of Gobiidae TLRs. [file 12915_2019_731_MOESM18_ESM.pdf]

Supplemental_Fig_S10
The round goby genome

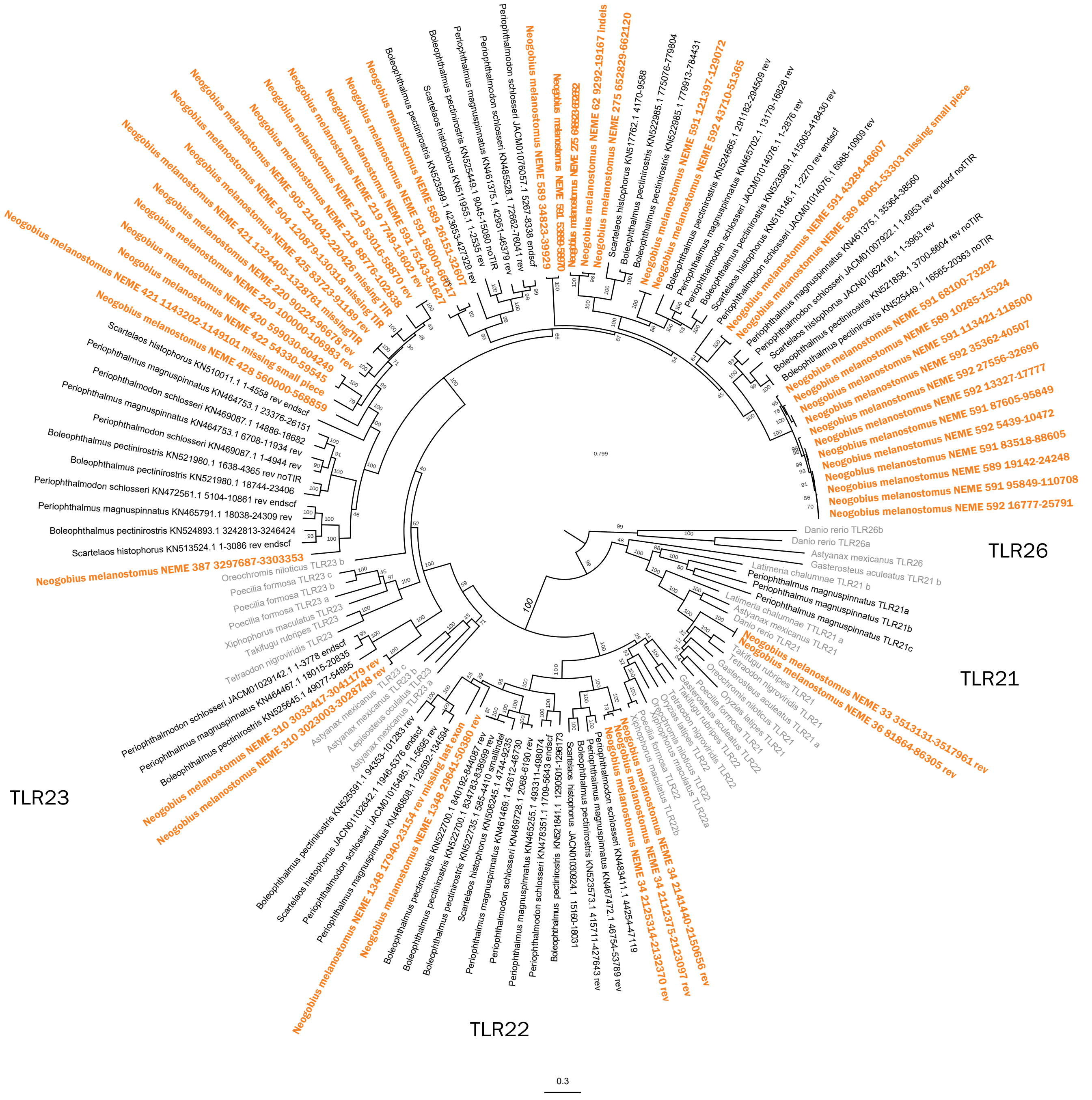

Phylogenetic tree of Gobiidae Toll Like Receptor protein sequences. A maximum likelihood phylogenetic tree run with the JTT substitution model and 500 bootstrap replicates on the transmembrane, linker and TIR domain of all TLRs found in Gobiiformes (Neogobius melanostomus, orange; other Gobiidae, black) and selected other fish (grey) for context.
