# Supplementary material for: The round goby genome provides insights into mechanisms that may facilitate biological invasions
Source: BMC Biol. 2020 Jan 28;18:11. doi: 10.1186/s12915-019-0731-8 (PMC6988351; doi:10.1186/s12915-019-0731-8)
Supplement: Supplementary file 19 — Figure S11. Phylogenetic tree of CRP / APCS. [file 12915_2019_731_MOESM19_ESM.pdf]

Supplemental_Fig_S11

The round goby genome

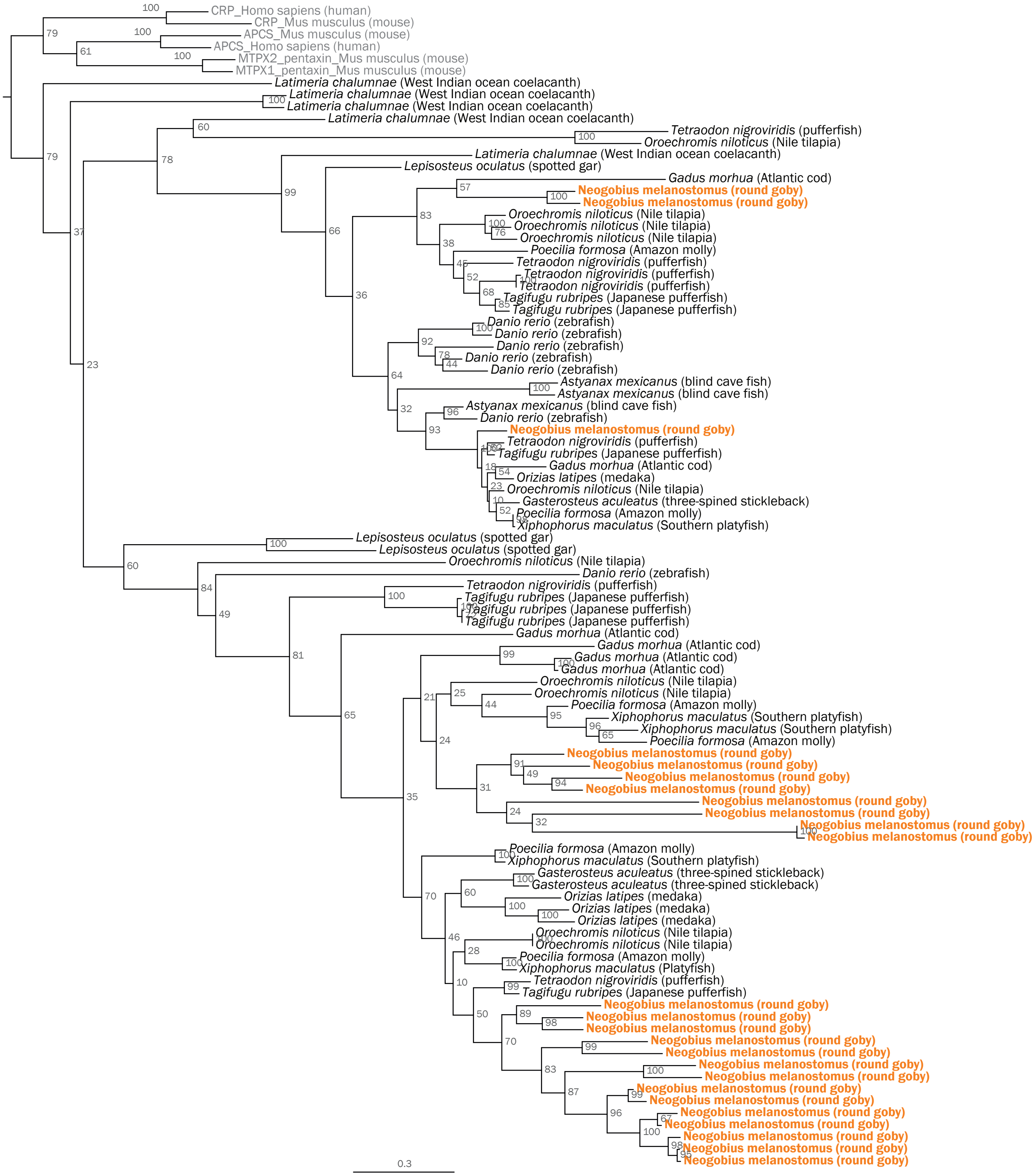

Phylogenetic tree of fish CRP/APCS sequences. Maximum Likelihood phylogenetic tree with 500 bootstraps rooted at the split between tetrapods and ray-finned fish. Tetrapods were used as outgroup and are indicated in grey. Round goby is indicated in orange.
