# Supplementary material for: The round goby genome provides insights into mechanisms that may facilitate biological invasions
Source: BMC Biol. 2020 Jan 28;18:11. doi: 10.1186/s12915-019-0731-8 (PMC6988351; doi:10.1186/s12915-019-0731-8)
Supplement: Supplementary file 20 — Figure S12. Phylogenetic trees of SUZ12, EED, and RBBP4. [file 12915_2019_731_MOESM20_ESM.pdf]

Supplemental_Fig_S12
The round goby genome

EED

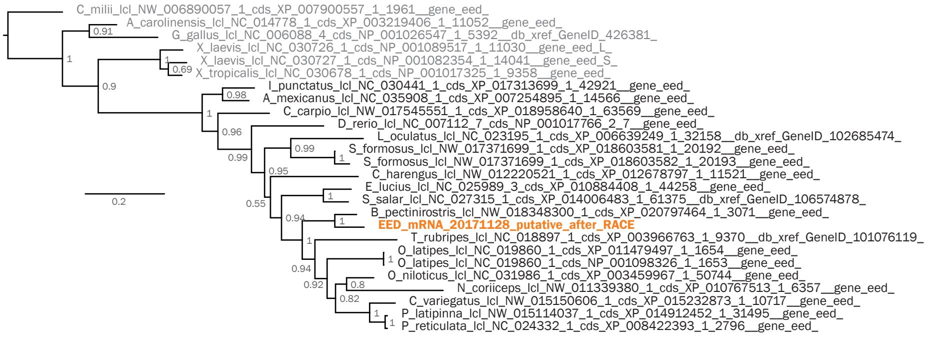

SUZ12

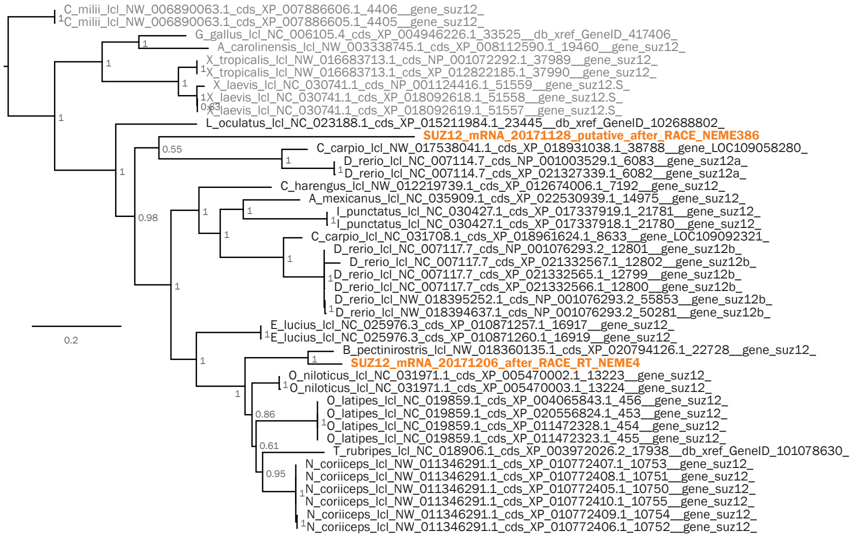

RBBP4

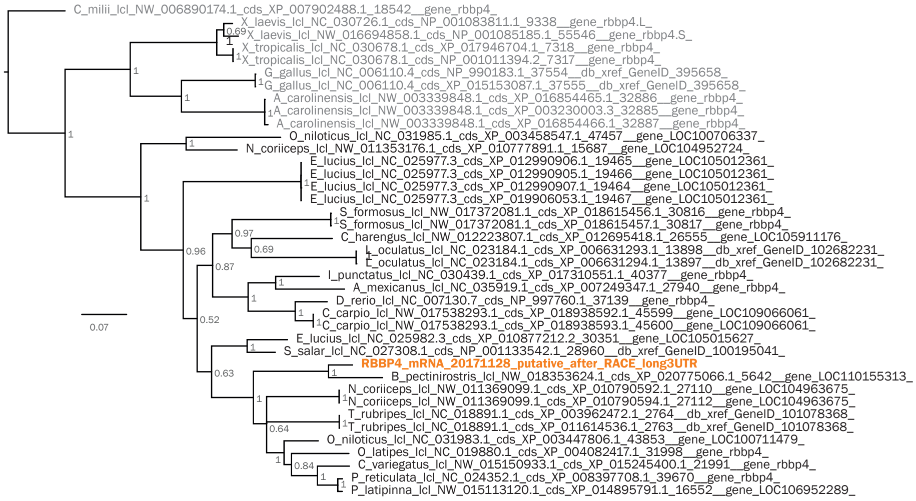

Phylogenetic tree of vertebrate PRC2 components EED, SUZ12, and RBBP4. Bayesian phylogenetic tree rooted with Australian ghostshark (C. milii). Round goby is indicated in orange.
